# Supplementary figures and images for: MITOL-dependent ubiquitylation negatively regulates the entry of PolγA into mitochondria
Source: PLoS Biol. 2021 Mar 3;19(3):e3001139. doi: 10.1371/journal.pbio.3001139 (PMC7959396; doi:10.1371/journal.pbio.3001139)

S1 Fig

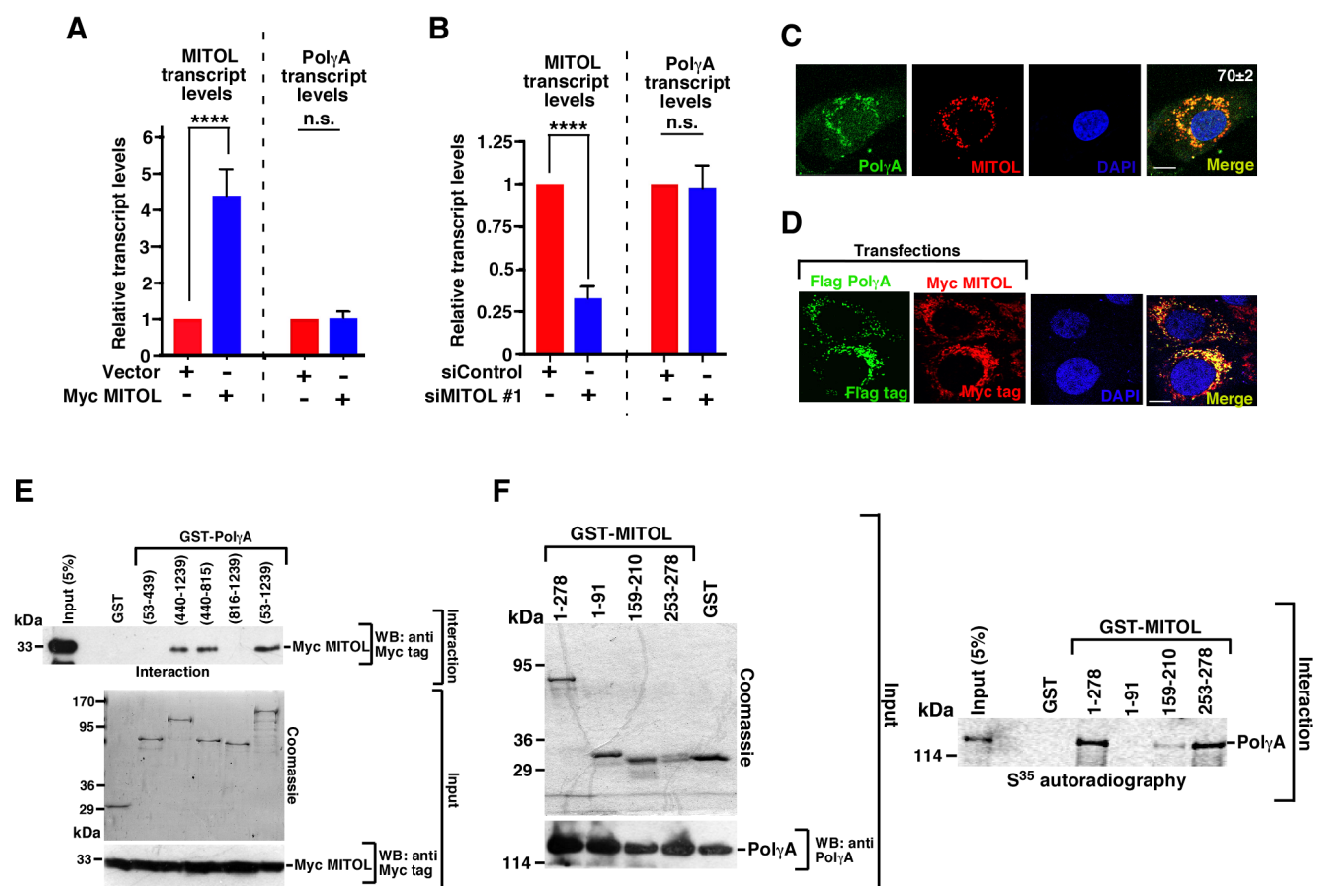

Supplement: S1 Fig — (A, B) Overexpression and ablation of MITOL does not alter PolγA transcript levels. HEK293T cells were either (A) transfected with either Myc MITOL or the corresponding vector or (B) transfected with either siControl or siMITOL RNA was isolated and RT-qPCR carried out to detect the levels of MITOL and PolγA. The transcript levels of GAPDH was used as control. (C) Endogenous PolγA colocalize with MITOL. Asynchronously growing NHFs were stained with antibodies against PolγA and MITOL. DNA was stained by DAPI. Scale, 5 μM. The colocalization factor has been indicated. Representative images are shown. (D) Overexpressed PolγA colocalize with exogenously expressed MITOL. U-2 OS cells were transfected with Flag PolγA WT and Myc MITOL. Cells were stained with anti-Flag and anti-Myc tag antibodies. Scale, 5 μM. Representative images are shown. (E) Spacer and thumb domains of PolγA interacts with MITOL. (Middle and bottom panels which constitute the input) Myc-MITOL expressed in HEK293T was detected by anti-Myc antibody and bound GST or GST-PolγA proteins [PolγA (53–439), PolγA (440–1239), PolγA (440–815), PolγA (816–1239), and PolγA (53–1239)] were detected by Coomassie staining. (Top) The interactions between Myc-MITOL and bound GST or GST-PolγA proteins were detected with anti-Myc tag antibody. Three independent biological replicates were carried out, and the same result was obtained. (F) Carboxyl terminus loop of MITOL interacts with PolγA. (Left panels, which constitute the input) PolγA (as visualized by western analysis of the S35 methionine radiolabeled in vitro transcribed and translated product with anti-PolγA antibody) and bound GST or GST-MITOL [MITOL (1–278), MITOL (1–191), MITOL (159–210), and MITOL (253–278)] were visualized by Coomassie staining. (Right) Interaction was carried out between S35 methionine radiolabeled PolγA and bound GST or GST-MITOL proteins. The amount of radiolabeled PolγA bound to the GST-tagged proteins was detected by autoradiography. Thre [file pbio.3001139.s001.pdf]

S2 Fig

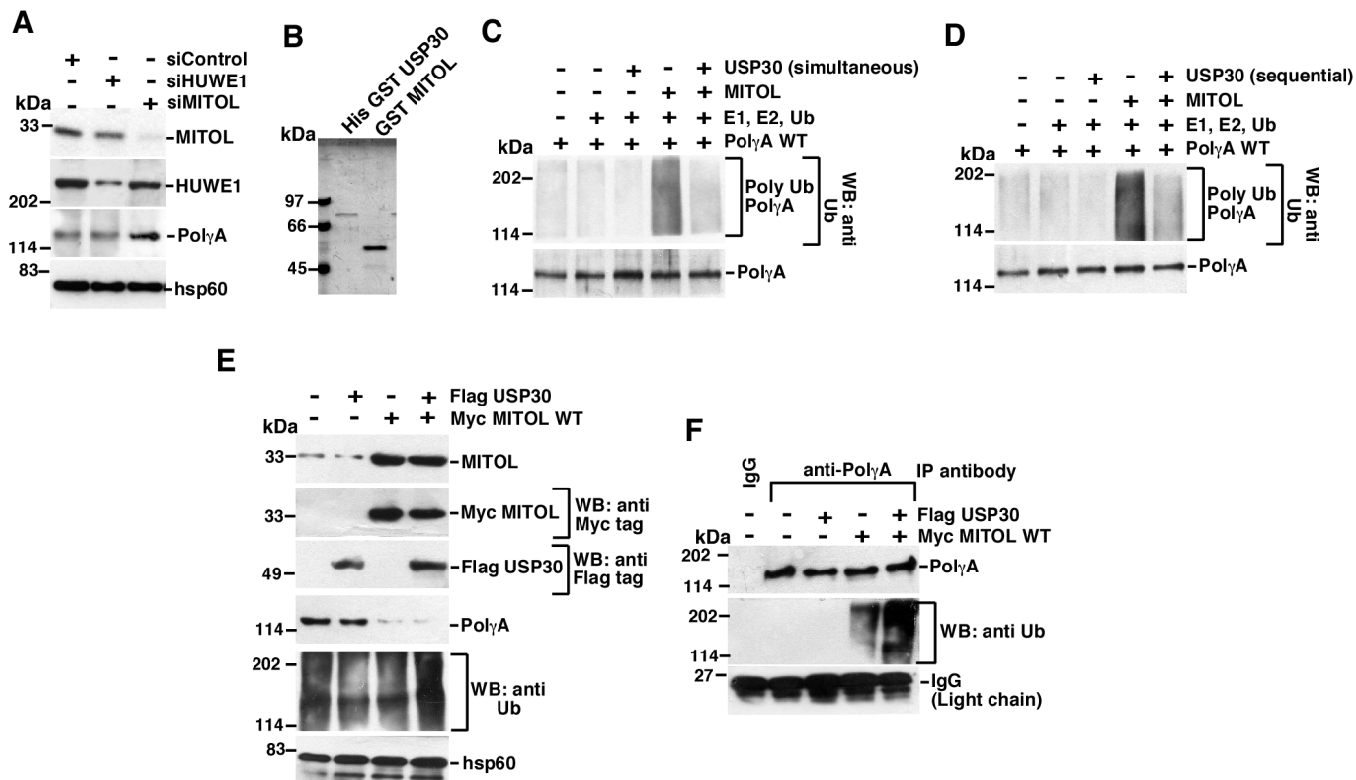

Supplement: S2 Fig — (A) PolγA is not a substrate of HUWE1. Lysates were made from HEK293T cells transfected with either siControl or siHUWEI or siMITOL. Western blot analysis was carried out with the indicated antibodies. Three independent experiments were done, and the same results were obtained. (B) Coomassie gel showing purified recombinant His GST USP30 and GST MITOL. Coomassie gels indicating the purity of His GST USP30 and GST-MITOL used in the assays. Three independent protein preparations were used for the experiments. (C, D) USP30 deubiquitylates PolγA in vitro. In vitro ubiquitylation reactions were carried out using PolγA as the substrate and MITOL WT. Recombinant USP30 was added either (C) during the in vitro ubiquitylation assay (called simultaneous) or (D) after MITOL-mediated in vitro ubiquitylation assay (called sequential). Post-reaction, the products were detected by western blot analysis with the indicated antibodies. Three biological replicates were carried out, and the same result was obtained. (E) Overexpression of USP30 cannot revert MITOL-mediated degradation of PolγA. Lysates were made from HEK293T cells transfected with either Flag USP30 or Myc MITOL WT. Western blot analysis was carried out with the indicated antibodies. Three independent experiments were done, and the same results were obtained. (F) Overexpression of USP30 cannot revert MITOL-mediated ubiquitylation of PolγA. Immunoprecipitations with either PolγA antibody (or the corresponding IgG) were carried out with lysates were made from HEK293T cells transfected with either Flag USP30 or Myc MITOL WT. Western blot analysis was carried out with the indicated antibodies. Three independent experiments were done, and the same results were obtained. IgG, immunoglobulin G; PolγA, polymerase γ subunit A; WT, wild-type. (PDF) [file pbio.3001139.s002.pdf]

**S3 Fig**

**A**

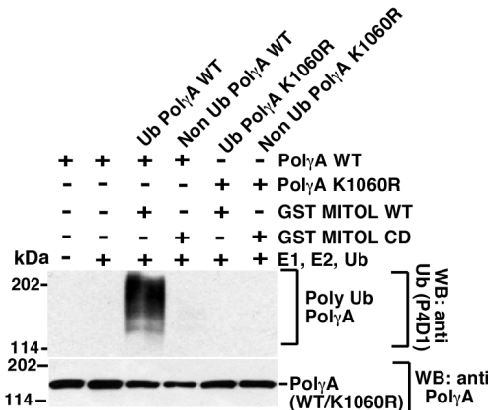

# B

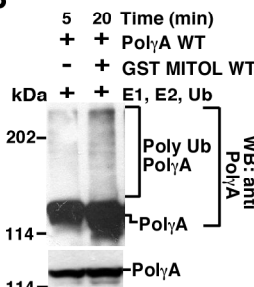

**C**

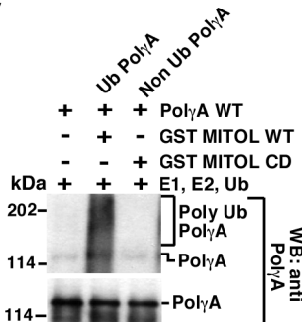

# D

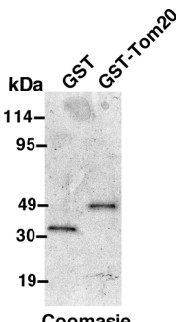

# E

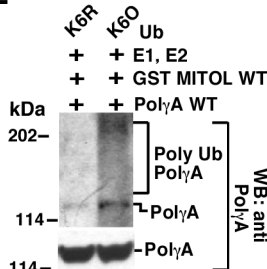

Supplement: S3 Fig — (A) Generation of ubiquitylated and non-ubiquitylated PolγA. MITOL WT or CD dependent in vitro ubiquitylation reactions were carried out with PolγA WT or K1060R. The ubiquitylated products were detected by carrying out western blot analysis with anti-Ub (P4D1) antibodies. Equal amounts of substrates used in each condition was determined by carrying out westerns with anti-PolγA antibodies. Four independent biological replicates were carried out, and the same result was obtained. (B) Time course of PolγA ubiquitylation by MITOL. In vitro ubiquitylation reactions were carried out using PolγA as the substrate and MITOL WT as the E3 ligase. (Top) The ubiquitylation reactions were carried out for 5 minutes and 20 minutes. Post-reaction, the products were detected by western blot analysis with anti-PolγA antibody. (Bottom) Pre-reaction, the amount of PolγA protein used in each ubiquitylation reaction was determined by western analysis using anti-PolγA antibodies. Three independent biological replicates were carried out, and the same result was obtained. (C) PolγA require catalytically active MITOL during in vitro ubiquitylation. In vitro ubiquitylation reactions were carried out using S35 methionine radiolabeled PolγA as the substrate and MITOL WT or CD as the E3 ligase. Post-ubiquitylation, the products were detected by carrying out western blot analysis with anti-PolγA antibodies. The reaction products where PolγA were ubiquitylated by MITOL WT were designated as Ub PolγA. Alternately, the products obtained when MITOL CD was used were designated as Non Ub PolγA. The amount of PolγA used in each condition was determined by western blotting with antibodies against PolγA. Three independent biological replicates were carried out, and the same result was obtained. (D) Purity of GST-Tom20. Coomassie gels indicating the purity of GST and GST-Tom20 used in the assays. Three independent protein preparations were used for the experiments. (E) PolγA was ubiquitylated by Ub K6O and [file pbio.3001139.s003.pdf]

S4 Fig

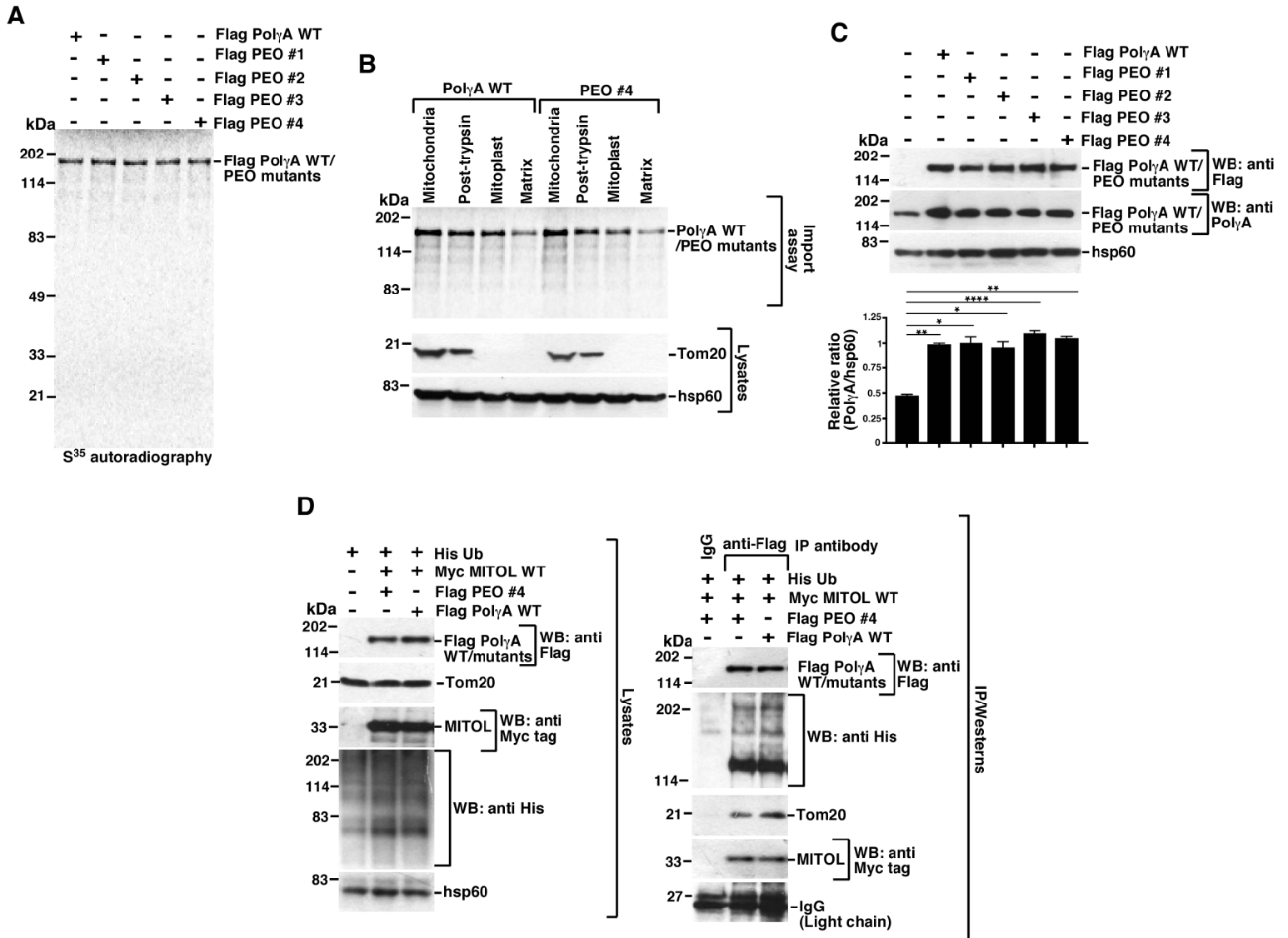

Supplement: S4 Fig — (A) Levels of PolγA WT and PEO mutants. In vitro transcribed and translated PolγA WT, PEO mutant #1, PEO mutant #2, PEO mutant #3, and PEO mutant #4 were subjected to SDS-PAGE, and the products were detected by autoradiography. Three independent biological replicates were carried out, and the same result was obtained. (B) PEO patient #4 shows similar extent of mitochondrial import as PolγA WT. Mitochondrial import assay was carried out using the indicated mitochondrial fractions. S35 methionine radiolabeled PolγA WT, PEO mutant #4 were incubated with each of the mitochondrial fractions. (Bottom) The purity of the mitochondrial fractions was determined using the indicated antibodies. Three independent biological replicates were carried out, and the same result was obtained. (C) Relative levels of endogenous and exogenous PolγA WT and PEO mutants. Lysates were made from HEK293T cells transfected with Flag PolγA WT, Flag PEO mutant #1, Flag PEO mutant #2, Flag PEO mutant #3, and Flag PEO mutant #4. Western blot analysis was carried out with the indicated antibodies. The relative levels of PolγA to hsp60 have been quantitated from 3 biological replicates. (D) PEO patient #4 shows similar extent of ubiquitylation and binding to Tom20 as PolγA WT. (Left) Whole cell extracts were made from HEK293T cells transfected with His-Ub, Myc MITOL WT, PolγA WT, and PEO mutant #4. Western blot analysis was carried out with the indicated antibodies. (Right) Immunoprecipitations were carried out with anti-Flag antibody (or the corresponding IgG), and the immunoprecipitates were probed with the indicated antibodies. Three independent biological replicates were carried out, and the same result was obtained. Numerical values for all graphs can be found in S1 Data. His-Ub, His-tagged ubiquitin; IgG, immunoglobulin G; PEO, progressive external ophthalmoplegia; PolγA, polymerase γ subunit A; WT, wild-type. (PDF) [file pbio.3001139.s004.pdf]

S5 Fig

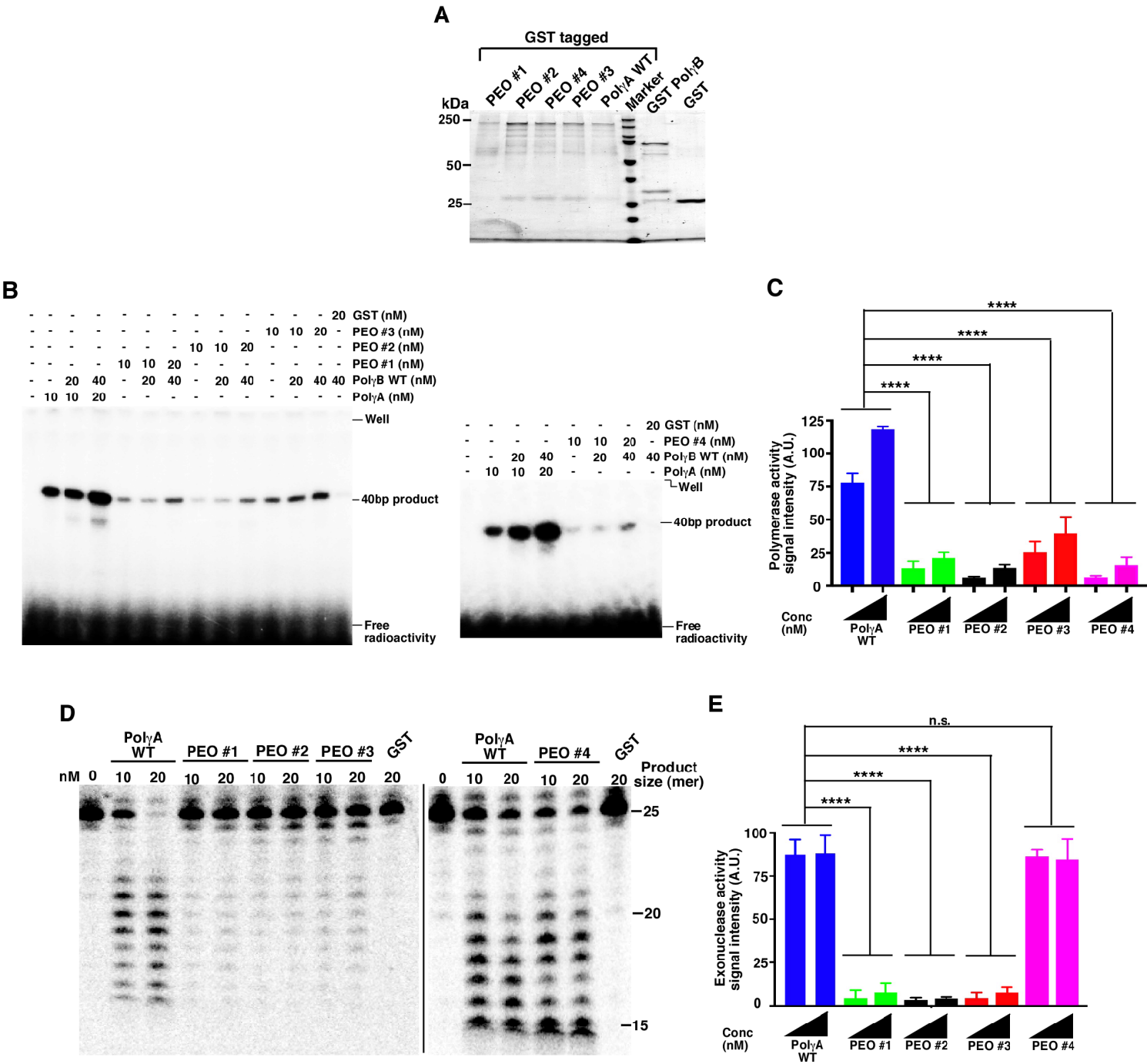

Supplement: S5 Fig — (A) Purity of proteins used in primer extension and exonuclease assays. Coomassie gels indicating the purity of GST-tagged PolγA WT, PEO mutant #1, PEO mutant #2, PEO mutant #3, PEO mutant #4, and GST-PolγB used in the primer extension and exonuclease assays. Two independent protein preparations were used for the experiments. (B, C) All PEO mutants show compromised in vitro polymerase activity. (B) In vitro primer extension assays were carried out with PolγA WT, PEO mutant #1, PEO mutant #2, PEO mutant #3, and PEO mutant #4 using the primer-template pair in presence of [α-P32] dATP. (C) The 40-bp radiolabeled product was quantitated from 3 biological replicates. (D, E) Except PEO mutant #4, all other PEO mutants show compromised in vitro exonuclease activity. (D) In vitro exonuclease assays were carried out with PolγA WT, PEO mutant #1, PEO mutant #2, PEO mutant #3, and PEO mutant #4 using the [γ-32P] ATP labeled primer annealed to the template. (E) The smaller products obtained were quantitated from 3 biological replicates. Numerical values for all graphs can be found in S1 Data. PEO, progressive external ophthalmoplegia; PolγA, polymerase γ subunit A; WT, wild-type. (PDF) [file pbio.3001139.s005.pdf]

S6 Fig

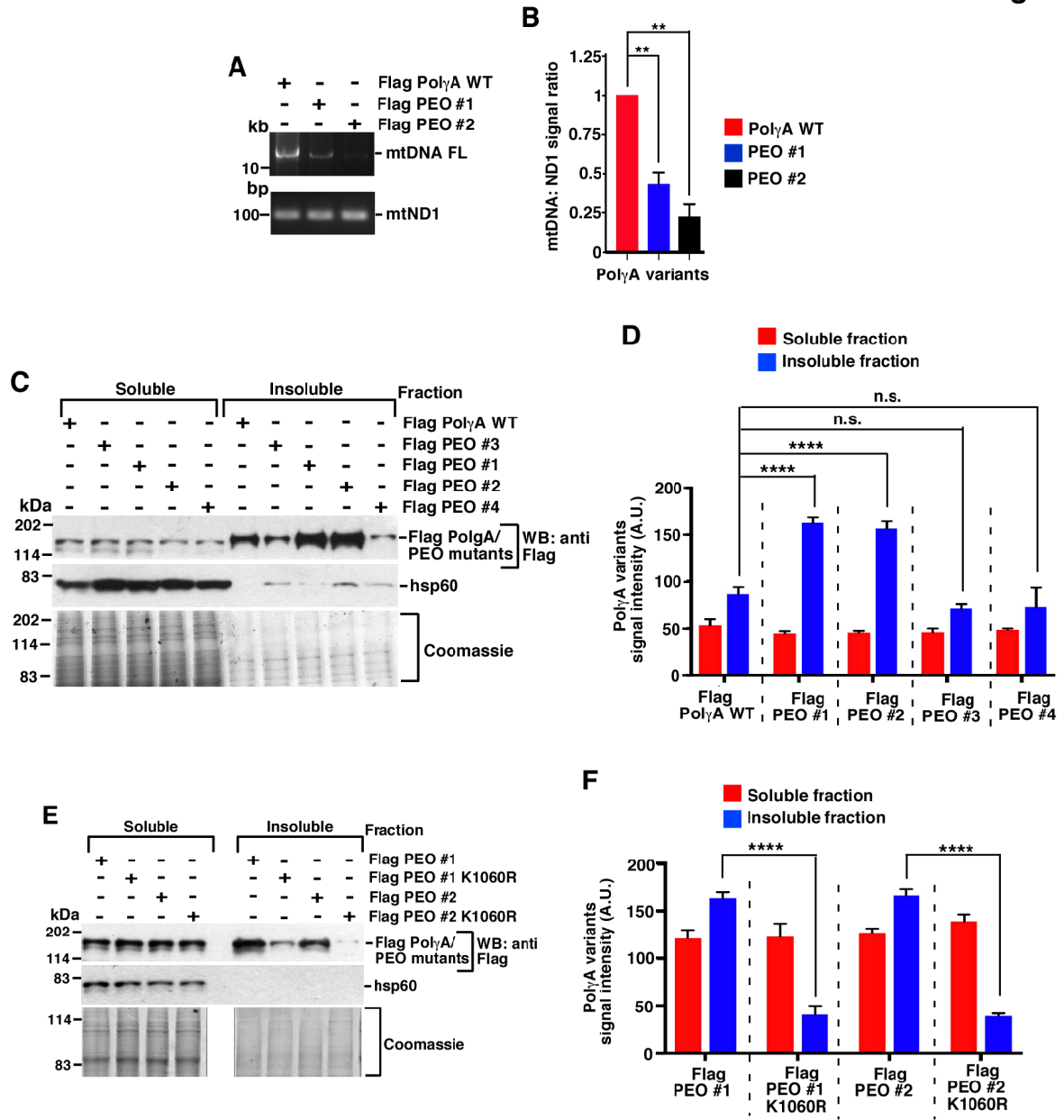

Supplement: S6 Fig — (A, B) Cells expressing PEO mutant #1 and #2 have lower mtDNA integrity. (A) mtDNA integrity was measured using a long-range mtDNA amplification assay in HEK293T cells expressing Flag PolγA WT, Flag PEO mutant #1, and Flag PEO mutant #2. Amplification of mtND1 was used as a control. (B) Quantitation of (A), done with data from 3 biological replicates. (C, D) Subsets of PEO mutants are present in the insoluble fraction. (C) Soluble and insoluble fractions were made from HEK293T cells overexpressing Flag PolγA WT, Flag PEO mutant #1, Flag PEO mutant #2, Flag PEO mutant #3, and Flag PEO mutant #4. Western blot analysis was carried out with the indicated antibodies. Equal amounts of the soluble and insoluble fractions were visualized by Coomassie staining. (D) Quantitation of (C), done with data from 3 biological replicates. (E, F) K1060R mutation in PEO mutants #1 and #2 leads to their decreased presence in the insoluble fraction. (E) Soluble and insoluble fractions were made from HEK293T cells overexpressing Flag PEO mutant #1, Flag PEO mutant #1 K1060R, Flag PEO mutant #2, and Flag PEO mutant #2 K1060R. Western blot analysis was carried out with the indicated antibodies. (F) Quantitation of (E), done with data from 3 biological replicates. Numerical values for all graphs can be found in S1 Data. mtDNA, mitochondrial DNA; PEO, progressive external ophthalmoplegia; PolγA, polymerase γ subunit A; WT, wild-type. (PDF) [file pbio.3001139.s006.pdf]
