# Supplementary material for: MITOL-dependent ubiquitylation negatively regulates the entry of PolγA into mitochondria
Source: PLoS Biol. 2021 Mar 3;19(3):e3001139. doi: 10.1371/journal.pbio.3001139 (PMC7959396; doi:10.1371/journal.pbio.3001139)
Supplement: S1 Table — (PDF) [file pbio.3001139.s007.pdf]

S1 Table: Details of patient mutations used in the study

PEO patient mutants		
**Identifier (lab reference)**	**Mutation**	**Approximate Molecular Weight**
PEO patient mutant #1	F961S	139kDa
PEO patient mutant #2	A467T	139kDa
PEO patient mutant #3	W748S	139kDa
PEO patient mutant #4	Y955C	139kDa
