# Supplementary material for: MITOL-dependent ubiquitylation negatively regulates the entry of PolγA into mitochondria
Source: PLoS Biol. 2021 Mar 3;19(3):e3001139. doi: 10.1371/journal.pbio.3001139 (PMC7959396; doi:10.1371/journal.pbio.3001139)
Supplement: S2 Table — (PDF) [file pbio.3001139.s008.pdf]

S2 Table: List of antibodies used in the study

Name of antibody	Source	Identifier
Anti-PolγA (used for WB)	Santa Cruz Biotechnology	Cat# sc-48815; RRID:AB_2166864
Anti-PolγA (used for WB)	Santa Cruz Biotechnology	Cat# sc-390634
Anti-PolγA (used for IF)	Santa Cruz Biotechnology	Cat# sc-5930; RRID:AB_2166868
Anti-MITOL (used for WB, IF)	Shigeru Yanagi (Tokyo University of Pharmacy and Life Sciences, Japan)	[1]
Anti-hsp60 (used for WB)	Abcam	Cat# ab87085; RRID:AB_10672924
Anti-Myc tag (used for WB, IF)	Cell Signaling Technology	Cat#2278; RRID:AB_10693332
Anti-Flag (used for WB, IF)	Sigma-Aldrich	Cat# F1804; RRID:AB_262044
Anti-Flag M2 affinity gel (used for IP)	Sigma-Aldrich	Cat# F2220
Anti-TFAM (used for WB)	Abcam	Cat# ab131607; RRID:AB_11154693
Anti-PolγB (used for WB)	Sigma-Aldrich	Cat# SAB1402537; RRID:AB_10640759
Anti-Twinkle (used for WB)	Abcam	Cat# ab83329; RRID:AB_1859960
Anti-Ub (P4D1) (used for WB)	Santa Cruz Biotechnology	Cat# sc-8017; RRID:AB_628423
Anti-His (used for WB)	Santa Cruz Biotechnology	Cat# sc-8036; RRID:AB_627727
Anti-Tom20 (used for WB)	Santa Cruz Biotechnology	Cat# sc-17764; RRID:AB_628381
Anti-BrdU (used for SBW, SW)	Abcam	Cat# ab1893; RRID: AB_302659
Anti-HUWE1 (used for WB)	Bethyl Laboratories	Cat# A300-486A; RRID: AB_ AB_2264590
Anti-diUbiquitin K6 affimer (used for WB)	Avacta	Cat# AVA00100

WB: Western blotting
IP: Immunoprecipitation
IF: Immunofluorescence
SB: Slot blot western
SW: Southwestern

References:

1. Yonashiro R, Ishido S, Kyo S, Fukuda T, Goto E, Matsuki Y, et al. A novel mitochondrial ubiquitin ligase plays a critical role in mitochondrial dynamics. EMBO J. 2006;25(15):3618-26. doi: 10.1038/sj.emboj.7601249. PubMed PMID: 16874301; PubMed Central PMCID: PMCPMC1538564.
