# Supplementary material for: MITOL-dependent ubiquitylation negatively regulates the entry of PolγA into mitochondria
Source: PLoS Biol. 2021 Mar 3;19(3):e3001139. doi: 10.1371/journal.pbio.3001139 (PMC7959396; doi:10.1371/journal.pbio.3001139)
Supplement: S3 Table — (PDF) [file pbio.3001139.s009.pdf]

S3 Table: List of recombinant DNAs used in the study

Name of the recombinant DNA	Source	Identifier
pA Puro MITOL WT-myc or Myc MITOL WT	Shigeru Yanagi (Tokyo University of Pharmacy and Life Sciences, Japan)	[1]
pA Puro MITOL CD-myc or Myc MITOL CD	This study	N/A
pGEX4T-1 MITOL WT or GST MITOL WT	This study	N/A
pGEX4T-1 MITOL CD or GST MITOL CD	This study	N/A
pGEX4T-1 MITOL N ter or GST MITOL (1-91)	Shigeru Yanagi (Tokyo University of Pharmacy and Life Sciences, Japan)	[2]
pGEX4T-1 MITOL 2nd loop or GST MITOL (159-210)	Shigeru Yanagi (Tokyo University of Pharmacy and Life Sciences, Japan)	[2]
pGEX4T-1 MITOL C ter or GST MITOL (253-278)	Shigeru Yanagi (Tokyo University of Pharmacy and Life Sciences, Japan)	[2]
pcDNA4-TO myc-his-B PARKIN WT	Quan Chen (State Key Laboratory of Membrane Biology, China)	[3]
pcDNA4-TO myc-his-B MULAN WT	Quan Chen (State Key Laboratory of Membrane Biology, China)	[3]
pFLAG-CMV4 RNF185 WT	Quan Chen (State Key Laboratory of Membrane Biology, China)	[3]
pFLAG-CMV4 KEAP1 WT	Quan Chen (State Key Laboratory of Membrane Biology, China)	[3]
His-Ub	Akhil Banerjea, (National Institute of Immunology, India)	N/A
pcDNA3.1 hygro(+)-Flag-PolγA WT	Present in the lab of corresponding author	[4]
pcDNA3.1 hygro(+)-Flag-PolγA (K981R)	This study	N/A

pcDNA3.1 hygro(+)-Flag-Polγ A (K990R)	This study	N/A
pcDNA3.1 hygro(+)-Flag-Polγ A (K1060R)	This study	N/A
pcDNA3.1 hygro(+)-Flag-Polγ A F961S or Flag PEO mutant #1	This study	N/A
pcDNA3.1 hygro(+)-Flag-Polγ A F961S K1060R or Flag PEO mutant #1 K1060R	This study	N/A
pcDNA3.1 hygro(+)-Flag-Polγ A A467T or Flag PEO mutant #2	This study	N/A
pcDNA3.1 hygro(+)-Flag-Polγ A A467T K1060R or Flag PEO mutant #2 K1060R	This study	N/A
pcDNA3.1 hygro(+)-Flag-Polγ A W748S or Flag PEO mutant #3	This study	N/A
pcDNA3.1 hygro(+)-Flag-Polγ A Y955C or Flag PEO mutant #4	This study	N/A
pcDNA 3.1 hygro (+) Polγ A WT	This study	N/A
pGEX4T-1 Polγ A (53-1239) or GST Polγ A WT	Present in the lab of corresponding author	[5]
pGEX4T-1 Polγ A (53-439) or GST Polγ A (53-439)	Present in the lab of corresponding author	[5]
pGEX4T-1 Polγ A (440-1239) or GST Polγ A (440-1239)	Present in the lab of corresponding author	[5]
pGEX4T-1 Polγ A (440-815) or GST Polγ A (440-815)	Present in the lab of corresponding author	[5]
pGEX4T-1 Polγ A (816-1239) or GST Polγ A (816-1239)	Present in the lab of corresponding author	[5]
pGEX4T-1 Polγ A F961S or GST PEO mutant #1	This study	N/A
pGEX4T-1 Polγ A A467T or GST PEO mutant #2	This study	N/A
pGEX4T-1 Polγ A W748S or GST PEO mutant #3	This study	N/A
pGEX4T-1 Polγ A Y955C or GST PEO mutant #4	This study	N/A
pGEX4T-1 Polγ B (27-485) or GST Polγ B WT	Present in the lab of corresponding author	[5]
pGEX-6P-1-ratTom20 (59–126)	Daisuke Kohda (Kyushu University, Japan)	[6]
Flag-HA-USP30	Wade Harper (Addgene plasmid #22578)	[7]
His-GST-USP30	David Komander (Addgene plasmid #110744)	[8]

References

1. Yonashiro R, Ishido S, Kyo S, Fukuda T, Goto E, Matsuki Y, et al. A novel mitochondrial ubiquitin ligase plays a critical role in mitochondrial dynamics. EMBO J. 2006;25(15):3618-26. doi: 10.1038/sj.emboj.7601249. PubMed PMID: 16874301; PubMed Central PMCID: PMCPMC1538564.
2. Sugiura A, Nagashima S, Tokuyama T, Amo T, Matsuki Y, Ishido S, et al. MITOL regulates endoplasmic reticulum-mitochondria contacts via Mitofusin2. Mol Cell. 2013;51(1):20-34. doi: 10.1016/j.molcel.2013.04.023. PubMed PMID: 23727017.
3. Chen Z, Liu L, Cheng Q, Li Y, Wu H, Zhang W, et al. Mitochondrial E3 ligase MARCH5 regulates FUNDC1 to fine-tune hypoxic mitophagy. EMBO Rep. 2017;18(3):495-509. Epub 2017/01/21. doi: 10.15252/embr.201643309. PubMed PMID: 28104734; PubMed Central PMCID: PMCPMC5331199.
4. De S, Kumari J, Mudgal R, Modi P, Gupta S, Futami K, et al. RECQL4 is essential for the transport of p53 to mitochondria in normal human cells in the absence of exogenous stress. J Cell Sci. 2012;125(Pt 10):2509-22. Epub 2012/02/24. doi: jcs.101501 [pii] 10.1242/jcs.101501. PubMed PMID: 22357944.
5. Gupta S, De S, Srivastava V, Hussain M, Kumari J, Muniyappa K, et al. RECQL4 and p53 potentiate the activity of polymerase gamma and maintain the integrity of the human mitochondrial genome. Carcinogenesis. 2014;35(1):34-45. Epub 2013/09/27. doi: bgt315 [pii]
10.1093/carcin/bgt315. PubMed PMID: 24067899.
6. Saitoh T, Igura M, Obita T, Ose T, Kojima R, Maenaka K, et al. Tom20 recognizes mitochondrial presequences through dynamic equilibrium among multiple bound states. Embo J. 2007;26(22):4777-87. PubMed PMID: 17948058.
7. Sowa ME, Bennett EJ, Gygi SP, Harper JW. Defining the human deubiquitinating enzyme interaction landscape. Cell. 2009;138(2):389-403. Epub 2009/07/21. doi: 10.1016/j.cell.2009.04.042. PubMed PMID: 19615732; PubMed Central PMCID: PMCPMC2716422.
8. Gersch M, Gladkova C, Schubert AF, Michel MA, Maslen S, Komander D. Mechanism and regulation of the Lys6-selective deubiquitinase USP30. Nature structural & molecular biology. 2017;24(11):920-30. Epub 2017/09/26. doi: 10.1038/nsmb.3475. PubMed PMID: 28945249; PubMed Central PMCID: PMCPMC5757785.
