# Supplementary material for: MITOL-dependent ubiquitylation negatively regulates the entry of PolγA into mitochondria
Source: PLoS Biol. 2021 Mar 3;19(3):e3001139. doi: 10.1371/journal.pbio.3001139 (PMC7959396; doi:10.1371/journal.pbio.3001139)
Supplement: S4 Table — (PDF) [file pbio.3001139.s010.pdf]

S4 Table: List of reagents used in the study

Name	Source	Identifier
**Chemicals**		
Cycloheximide	Sigma-Aldrich	Cat# C7698; CAS Number 66-81-9
MG132	Merck	Cat# 474787; CAS Number 133407-82-6
4-Hydroxytamoxifen	Sigma-Aldrich	Cat# H7904; CAS Number 68047-06-3
IPTG	Sigma-Aldrich	Cat# I6758; CAS Number 367-93-1
PMSF	Sigma-Aldrich	Cat# P7626; CAS Number 329-98-6
DTT	Sigma-Aldrich	Cat# D0632; CAS Number 3483-12-3
BrdU	Sigma-Aldrich	Cat# B5002; CAS Number 59-14-3
Triton-X-100	Sigma-Aldrich	Cat# T9284; CAS Number 9002-93-1
CCCP	Sigma-Aldrich	Cat# C2759; CAS Number 555-60-2
**Recombinant proteins**		
Ubiquitin	Enzo	Cat# BML-UW8795-0005; CAS Number 79586
Ubiquitin activating enzyme E1 Ube 1 (human)	Enzo	Cat# BML-UW9410-0050
UbcH5a (human)	Enzo	Cat# BML-UW9050-0100
Ubiquitin (K63O)	Boston Biochem	Cat# UM-HK630
Ubiquitin (K48O)	Boston Biochem	Cat# UM-HK480
Ubiquitin (K6R)	Boston Biochem	Cat# UM-K6R
Ubiquitin (K11R)	Boston Biochem	Cat# UM-K11R
Ubiquitin (K27R)	Boston Biochem	Cat# UM-K27R
Ubiquitin (K29R)	Boston Biochem	Cat# UM-K29R
Ubiquitin (K33R)	Boston Biochem	Cat# UM-K33R
Ubiquitin (K48R)	Boston Biochem	Cat# UM-K48R
Ubiquitin (K63R)	Boston Biochem	Cat# UM-K63R
Ubiquitin No K	Boston Biochem	Cat# UB-NOK
GST MITOL Wildtype (WT)	This study	N/A
GST MITOL Catalytic Dead (CD)	This study	N/A
GST Tom20	This study	N/A
GST PolγA WT	This study	N/A
GST PEO mutant #1	This study	N/A
GST PEO mutant #2	This study	N/A
GST PEO mutant #3	This study	N/A
GST PEO mutant #4	This study	N/A
GST PolγB WT	This study	N/A
GST PolγA (53-439)	This study	N/A
GST PolγA (440-1239)	This study	N/A
GST PolγA (440-815)	This study	N/A
GST PolγA (816-1239)	This study	N/A
GST MITOL (1-91)	This study	N/A

GST MITOL (159-210)	This study	N/A
GST MITOL (253-278)	This study	N/A
**Cell Lines**		
HEK293T	Present in the lab of corresponding author	ATCC Cat# CRL-3216
HeLa shGFP	Shigeru Yanagi (Tokyo University of Pharmacy and Life Sciences, Japan)	[1]
HeLa shMITOL	Shigeru Yanagi (Tokyo University of Pharmacy and Life Sciences, Japan)	[1]
MITOL$^{flox/flox}$ MEFs	Shigeru Yanagi (Tokyo University of Pharmacy and Life Sciences, Japan)	[1]
GM07532-hTERT (Normal Human Fibroblasts, NHF)	Present in the lab of corresponding author	[2]
U-2 OS	Present in the lab of corresponding author	ATCC Cat# HTB-96
**Oligonucleotides**		
siRNA sequences for MITOL	Dharmacon	[3]
siRNA sequences for HUWE1	Dharmacon	[4]
siRNA sequences for Tom20	Dharmacon	Cat# L-006487-01
ON-TARGETplus Non-targeting siRNA #1	Dharmacon	Cat # D-001810-01-05
Primers for Long range mtDNA amplification assay, see Table S5	Sigma-Aldrich	N/A
**Other**		
Fetal bovine Serum	Thermo Fisher Scientific	Cat# 10082147
Advanced DMEM	Thermo Fisher Scientific	Cat# 12491-023
QuikChange II XL Site-Directed Mutagenesis Kit	Agilent	Cat# 200522
Mitochondrial DNA isolation kit	BioVision, Inc.	Cat# K280-50

T7 Quick coupled Transcription/ Translation system	Promega	Cat# L2080
[^{35}S] Methionine	Perkin Elmer	Cat# NEG009T
[α-^{32}P] dATP	Perkin Elmer	Cat# NEG012H
[γ-^{32}P] ATP	Perkin Elmer	Cat# NEG035C
Trizol reagent	Thermo Fisher Scientific	Cat# 15596026
Reverse Transcriptase Core Kit	Eurogentec	Cat# RT-RTCK-05
Qubit dsDNA HS assay kit	Thermo Fisher Scientific	Cat# Q32851
Lipofectamine 2000	Thermo Fisher Scientific	Cat# 11668019
Complete Protease Cocktail inhibitor	Roche	Cat# 11697498001
BL21-CodonPlus-RP	Agilent	Cat# 230250
Poly-Prep Chromatography column	Biorad	Cat# 73101550
Flag peptide	Sigma-Aldrich	Cat# 3290
LongAmp Taq DNA polymerase	New Englands Biolab	Cat# M0323L
QIAamp DNA Mini Kit	Qiagen	Cat# 51304
Immobilon Western Chemiluminescent HRP substrate	Merck	Cat# WBKLS0500

Reference:

1. Sugiura A, Nagashima S, Tokuyama T, Amo T, Matsuki Y, Ishido S, et al. MITOL regulates endoplasmic reticulum-mitochondria contacts via Mitofusin2. Mol Cell. 2013;51(1):20-34. doi: 10.1016/j.molcel.2013.04.023. PubMed PMID: 23727017.
2. Sengupta S, Linke SP, Pedeux R, Yang Q, Farnsworth J, Garfield SH, et al. BLM helicase-dependent transport of p53 to sites of stalled DNA replication forks modulates homologous recombination. Embo J. 2003;22(5):1210-22. PubMed PMID: 12606585.
3. Yonashiro R, Ishido S, Kyo S, Fukuda T, Goto E, Matsuki Y, et al. A novel mitochondrial ubiquitin ligase plays a critical role in mitochondrial dynamics. EMBO J. 2006;25(15):3618-26. doi: 10.1038/sj.emboj.7601249. PubMed PMID: 16874301; PubMed Central PMCID: PMCPMC1538564.
4. Peter S, Bultinck J, Myant K, Jaenicke LA, Walz S, Muller J, et al. Tumor cell-specific inhibition of MYC function using small molecule inhibitors of the HUWE1 ubiquitin ligase. EMBO Mol Med. 2014;6(12):1525-41. Epub 2014/09/26. doi: 10.15252/emmm.201403927. PubMed PMID: 25253726; PubMed Central PMCID: PMCPMC4287973.
