# Supplementary material for: MITOL-dependent ubiquitylation negatively regulates the entry of PolγA into mitochondria
Source: PLoS Biol. 2021 Mar 3;19(3):e3001139. doi: 10.1371/journal.pbio.3001139 (PMC7959396; doi:10.1371/journal.pbio.3001139)
Supplement: S5 Table — (PDF) [file pbio.3001139.s011.pdf]

S5 Table: List of primers used in the study

RT-qPCR primers			
**Identifier**	**Forward primer (5' to 3')**	**Reverse primer (5' to 3')**	**Reference**
Human PolγA	GGA AGT CAC AGT GGA AGA AG	CAC ACG GCT GGT CAT AAA	This study
Human MITOL	CAG ATC ATG TCT CTG CTA CTC	GGT GTG CCT GTC GTA AAT	
Human GAPDH	GTC TCC TCT GAC TTC AAC AGC G	ACC ACC CTG TTG CTG TAG CCA A	
**Long range mtDNA amplification assay**			
mtND1	CAC CCA AGA ACA GGG TTT GT	TGG CCA TGG GTA TGT TGT TAA	[1]
mtDNA	TGA GGC CAA ATA TCA TTC TGA GGG GC	TTT CAT CAT GCG GAG ATG TTG GAT GG	
**Primer extension assay primers**			
**Identifier**	**Primer sequence (5' to 3')**		**Reference**
20-mer primer	CAG ATA CTG CGA CAT AGG GT		[2, 3]
40-mer template	GTC TAT GAC GCT GTA TCC CAC GAG GCC GAG GTC GCA GAG C		
**Exonuclease assay primers**			
**Identifier**	**Primer sequence (5' to 3')**		**Reference**
25-mer primer	GCC TCG CAG CCG TCC AAC CAA TGT T		[3, 4]
45-mer template	GGA CGG CAT TGG ATC GAG GTT GAG TTG GTT GGA CGG CTG CGA GGC		

Reference:

1. Chi Z, Nie L, Peng Z, Yang Q, Yang K, Tao J, et al. RecQL4 cytoplasmic localization: implications in mitochondrial DNA oxidative damage repair. Int J Biochem Cell Biol. 2012;44(11):1942-51. Epub 2012/07/25. doi: S1357-2725(12)00255-5 [pii] 10.1016/j.biocel.2012.07.016. PubMed PMID: 22824301; PubMed Central PMCID: PMC3461334.
2. Achanta G, Sasaki R, Feng L, Carew JS, Lu W, Pelicano H, et al. Novel role of p53 in maintaining mitochondrial genetic stability through interaction with DNA Pol gamma. EMBO J. 2005;24(19):3482-92. Epub 2005/09/16. doi: 7600819 [pii] 10.1038/sj.emboj.7600819. PubMed PMID: 16163384; PubMed Central PMCID: PMC1276176.
3. Gupta S, De S, Srivastava V, Hussain M, Kumari J, Muniyappa K, et al. RECQL4 and p53 potentiate the activity of polymerase gamma and maintain the integrity of the human mitochondrial genome. Carcinogenesis. 2014;35(1):34-45. Epub 2013/09/27. doi: bgt315 [pii]
10.1093/carcin/bgt315. PubMed PMID: 24067899.
4. Johnson AA, Johnson KA. Exonuclease proofreading by human mitochondrial DNA polymerase. J Biol Chem. 2001;276(41):38097-107. Epub 2001/07/31. doi: 10.1074/jbc.M106046200
M106046200 [pii]. PubMed PMID: 11477094.
