# Supplementary material for: MITOL-dependent ubiquitylation negatively regulates the entry of PolγA into mitochondria
Source: PLoS Biol. 2021 Mar 3;19(3):e3001139. doi: 10.1371/journal.pbio.3001139 (PMC7959396; doi:10.1371/journal.pbio.3001139)
Supplement: S6 Table — (PDF) [file pbio.3001139.s012.pdf]

S6 Table: Statistical analysis performed in this study

Fig number	Statistical Analysis performed
Fig 1A	One-way ANOVA
Fig 1C	One-way ANOVA
Fig 1D	One-way ANOVA
Fig 1E	One-way ANOVA
Fig 1F	Paired t test
Fig 1G	Paired t test
Fig 1I	Two-way ANOVA
Fig 3B	Two-way ANOVA
Fig 3D	Two-way ANOVA
Fig 3F	Two-way ANOVA
Fig 3H	Paired t test
Fig 3I	Paired t test
Fig 4B	Two-way ANOVA
Fig 4E	One-way ANOVA
Fig 4G	Two-way ANOVA
Fig 5A	One-way ANOVA
Fig 5D	Two-way ANOVA
Fig 6C	Two-way ANOVA
Fig 6E	Two-way ANOVA
Fig S1A	Two-way ANOVA
Fig S1B	Two-way ANOVA
Fig S4C	One-way ANOVA
Fig S5C	Two-way ANOVA
Fig S5E	Two-way ANOVA
Fig S6B	One-way ANOVA
Fig S6D	Two-way ANOVA
Fig S6F	Two-way ANOVA
