# Supplementary material for: MITOL-dependent ubiquitylation negatively regulates the entry of PolγA into mitochondria
Source: PLoS Biol. 2021 Mar 3;19(3):e3001139. doi: 10.1371/journal.pbio.3001139 (PMC7959396; doi:10.1371/journal.pbio.3001139)
Supplement: S1 Data — All numerical raw data are combined in a single PDF file, “S1_Data.” The presented data underlays Figs 1ACDEFGI, 3BDFHI, 4BEG, 5AD, and 6CE and S1AB, S4C, S5CE, and S6BDF Figs. (PDF) [file pbio.3001139.s013.pdf]

Fig 1A

	PolGA			hsp90			PolGA/hsp90		
	Expt #1	Expt #2	Expt #3	Expt #1	Expt #2	Expt #3	Expt #1	Expt #2	Expt #3
Untransfected	72.3	114.7	95.3	129.6	132.7	145.7	0.55787037	0.86435569	0.65408373
Myc Mitol	6	7.9	4.5	135.7	147.2	150.9	0.04421518	0.05366848	0.02982107
Myc Parkin	73.3	115.2	96.9	121.1	119	120.2	0.60528489	0.96806723	0.80615641
Myc Mulan	70.7	113.7	93	132.6	144	138.4	0.5331825	0.78958333	0.67196532
Flag RNF185	74.3	114.4	96.3	145.4	146.3	151.2	0.51100413	0.78195489	0.63690476
Flag Keap1	73.1	113.7	96	146.7	155.5	119	0.49829584	0.73118971	0.80672269

Fig 1C

	PolGA			hsp90			PolGA/hsp90		
	Expt #1	Expt #2	Expt #3	Expt #1	Expt #2	Expt #3	Expt #1	Expt #2	Expt #3
Untransfected	96.4	124.3	134.4	156.7	140	137.5	0.61518826	0.88785714	0.97745455
Myc Mitol	8.8	17.9	21.2	155	149.2	132.7	0.05677419	0.11997319	0.15975885
Myc Mitol+MG132	78.4	86.6	108.5	160.1	143.2	134.6	0.48969394	0.6047486	0.80609212

Fig 1D

	PolGA			hsp90			PolGA/hsp90		
	Expt #1	Expt #2	Expt #3	Expt #1	Expt #2	Expt #3	Expt #1	Expt #2	Expt #3
Untransfected	76	72.3	127.9	126.3	137.3	148.3	0.60174188	0.52658412	0.862441
Myc Mitol WT	4.3	3.7	6.5	129.2	132.5	149.4	0.03328173	0.02792453	0.04350736
Myc Mitol CD	82.4	76.6	111.5	125	133.8	145.6	0.6592	0.57249626	0.7657967

Fig 1E

	PolGA			hsp90			PolGA/hsp90		
	Expt #1	Expt #2	Expt #3	Expt #1	Expt #2	Expt #3	Expt #1	Expt #2	Expt #3
siControl	13.79	14.56	26.6	127.2	155.6	130.3	0.10841195	0.09357326	0.20414428
siMITOL #1	72.34	92.8	66.6	133.7	162.1	133	0.54106208	0.57248612	0.50075188
siMITOL #2	68.4	74.64	92.6	132	166.3	127.2	0.51818182	0.44882742	0.72798742

Fig 1F

	PolGA			hsp90			PolGA/hsp90		
	Expt #1	Expt #2	Expt #3	Expt #1	Expt #2	Expt #3	Expt #1	Expt #2	Expt #3
HeLa shGFP	64	78.5	65.76	144.7	131.2	155	0.4422944	0.59832317	0.42425806
HeLa shMITOL	146.3	176.38	172.3	147.6	129.2	152.4	0.99119241	1.36517028	1.13057743

Fig 1G

	PolGA			hsp90			PolGA/hsp60		
	Expt #1	Expt #2	Expt #3	Expt #1	Expt #2	Expt #3	Expt #1	Expt #2	Expt #3
MITOL f/f MEFs -OHT	66.7	78.5	52.7	123.1	149.5	140.2	0.54183591	0.52508361	0.37589158
MITOL f/f MEFs +OHT	222.5	242.8	190.2	125	147.8	144.9	1.78	1.64276049	1.3126294

Fig 1I

	siControl+CHX			Adjested siControl+CHX		
Time	Expt #1	Expt #2	Expt #3	Expt #1	Expt #2	Expt #3
0	101.3	116.7432	99.3147	100	100	100
3	17.4848561	55.5382892	33.9696099	17.26047	47.57304	34.20401
6	10.5461607	17.662966	21.4039665	10.41082	15.12976	21.55166
12	16.6295397	32.0795721	34.3242627	16.41613	27.47875	34.56111
24	21.1582474	30.5573925	18.1215858	20.88672	26.17488	18.24663

	siMitol+CHX			Adjested siMitol+CHX		
Time	Expt #1	Expt #2	Expt #3	Expt #1	Expt #2	Expt #3
0	126.2453	127.5634	119.6463	100	100	100
3	88.8022317	111.581326	82.7026051	70.34102	87.47127	64.83255
6	77.903677	88.2267636	72.2117783	61.70818	69.16307	56.60854
12	67.9391228	84.783432	63.6510623	53.81517	66.46376	49.89759
24	61.9924516	90.2920151	59.8190961	49.10476	70.78207	46.89362

Time	Adjusted siControl+CHX			Adjusted siMitol+CHX		
	Expt #1	Expt #2	Expt #3	Expt #1	Expt #2	Expt #3
0	100	100	100	100	100	100
3	17.26047	47.57304	34.20401	70.34102	87.47127	64.83255
6	10.41082	15.12976	21.55166	61.70818	69.16307	56.60854
12	16.41613	27.47875	34.56111	53.81517	66.46376	49.89759
24	20.88672	26.17488	18.24663	49.10476	70.78207	46.89362

Fig 3B

	Untagged PolGA WT intensity			Flag PolGA WT intensity		
	Expt #1	Expt #2	Expt #3	Expt #1	Expt #2	Expt #3
Mitochondria	109.4	102.776	110.749	112.818	113.763	93.698
Post-trypsin	63.605	78.887	70.378	56.079	70.585	59.068
Mitoplast	52.91	50.789	45.564	52.7	50.711	55.188
CCCP	4.6	2.2	6.05	4.65	5.7	4.15
Triton X-100	2.05	2.7	4.1	4.28	3.85	4.44
Matrix	49.55	54.82	43.35	55.74	67.75	55.85

Fig 3D

	Non Ub PolGA WT intensity			Ub PolGA WT intensity		
	Expt #1	Expt #2	Expt #3	Expt #1	Expt #2	Expt #3
Mitochondria	121.68	110.8	124.64	111.9	115.3	103.45
Post-trypsin	99.6	92.9	86.55	26.82	20.82	29.08
Mitoplast	82.1	90.85	95.4	22.7	30.7	35.865
CCCP	6.45	8.15	4.55	1.5	1.05	2.02
Triton X-100	8.35	5.7	9.1	1.85	0.95	3.4
Matrix	89.5	94.76	103.95	15.05	7.5	10.75

Fig 3F

	Non-Ub PolgA WT intensity			Ub PolgA WT intensity			Non-Ub K1060R intensity			Ub-K1060R intensity		
	Expt #1	Expt #2	Expt #3	Expt #1	Expt #2	Expt #3	Expt #1	Expt #2	Expt #3	Expt #1	Expt #2	Expt #3
Mitochondria	40.886	78.984	61.313	78.554	94.627	69	109.4	92.776	69.749	112.818	113.763	73.698
Trypsin	18.631	54.513	36.649	0.44	15.796	11.669	63.605	78.887	49.378	56.079	90.585	59.068
Mitoplast	14.643	68.452	42.29	-0.151	13.309	11.085	36.91	73.789	45.564	42.7	80.711	40.188

Fig 3H

	Signal intensity		
	Expt #1	Expt #2	Expt #3
Ub PolGA	155	167.4	212
Non Ub PolGA	43	37	89.6

	Relative signal intensity		
	Expt #1	Expt #2	Expt #3
Ub PolGA	1	1	1
Non Ub PolGA	0.27741935	0.22102748	0.42264151

Fig 3I

	Signal intensity		
	Expt #1	Expt #2	Expt #3
Ub K6R	193.7	254	237.6
Ub K6O	63.7	88.9	72.5

	Relative signal intensity		
	Expt #1	Expt #2	Expt #3
Ub K6R	1	1	1
Ub K6O	0.32885906	0.35	0.30513468

Fig 4B

	PolGA WT			PEO #1			PEO #2			PEO #3			PEO #4		
	Expt #1	Expt #2	Expt #3	Expt #1	Expt #2	Expt #3	Expt #1	Expt #2	Expt #3	Expt #1	Expt #2	Expt #3	Expt #1	Expt #2	Expt #3
Mitochondria	97.319	146.441	53.341	99.511	124.72	95.119	89.589	129.203	73.269	99.134	174.847	69.053	76.313	72.146	72.654
Post trypsin	92.278	82.388	44.143	42.904	19.783	10.278	27.179	37.709	4.092	95.458	103.923	80.38	59.589	58.213	37.309
Mitoplast	51.85	63.709	26.865	3.565	0.812	3.073	5.108	13.418	2.525	83.314	102.217	27.065	49.195	46.023	24.583
Matrix	58.247	52	22.635	3.989	-0.329	1.101	4.346	4.584	2.415	53.041	67.253	16.199	31.185	23.066	18.334

Fig 4E

	PolGA			hsp90			PolGA/hsp90		
	Expt #1	Expt #2	Expt #3	Expt #1	Expt #2	Expt #3	Expt #1	Expt #2	Expt #3
PolGA WT	98.6	90.2	102.4	132.6	146.7	172.5	0.74358974	0.61486026	0.59362319
PolGA WT + MITOL WT	111.8	89.6	97.8	140	148.2	144.5	0.79857143	0.60458839	0.67681661
PolGA WT + MITOL WT + MG132	95.5	87.8	98.9	146.6	148.6	138.7	0.65143247	0.59084791	0.71304975
PEO #1	88.7	99.9	101.7	132.7	156.3	144.1	0.66842502	0.63915547	0.70575989
PEO #1 + MITOL WT	38.8	20.2	25.7	145.4	140.2	136	0.26685007	0.14407989	0.18897059
PEO #1 + MITOL WT + MG132	90.4	87.3	96.6	150	144	129	0.60266667	0.60625	0.74883721
PEO #2	107.7	100.6	115.1	140.8	138.2	144.8	0.76491477	0.72793054	0.7948895
PEO #2 + MITOL WT	26.4	22.8	20.8	130	139.4	142	0.20307692	0.16355811	0.14647887
PEO #2 + MITOL WT + MG132	88.6	89	102.3	146.5	132	134	0.60477816	0.67424242	0.76343284
PEO #3	92.5	99.4	90.1	140.5	135.3	129.4	0.65836299	0.73466371	0.69629057
PEO #3 + MITOL WT	95.4	96.5	98.9	142.5	144	139.8	0.66947368	0.67013889	0.7074392
PEO #3 + MITOL WT + MG132	103.2	97.7	94.6	146.3	145.2	142.1	0.70539986	0.67286501	0.66572836

Fig 4G

	Slot Blot					Southwestern				
	PolGA WT	PEO #1	PEO #2	PEO #3	PEO #4	PolGA WT	PEO #1	PEO #2	PEO #3	PEO #4
Expt #1	68.258	15.42	15.502	52.696	54.988	88.836	18.92	16.4	72.3	68.9
Expt #2	68.157	10.459	7.457	47.761	60.724	78.79	16.44	17.4	70.4	74.12
Expt #3	54.621	8.416	12.399	57.268	50.313	74.5	10.6	19.9	60.35	66.03

Fig 5A

	PolGA			hsp90			PolGA/hsp90		
	Expt #1	Expt #2	Expt #3	Expt #1	Expt #2	Expt #3	Expt #1	Expt #2	Expt #3
HeLa shGFP PolGA WT	106.2	95.6	94.6	119	132.5	133.2	0.89243697	0.72150943	0.71021021
HeLa shGFP PEO #1	7.3	7.8	14.6	107.6	137.3	130.4	0.06784387	0.05680991	0.11196319
HeLa shGFP PEO #2	6.8	5.6	11.9	113.6	130.7	138.7	0.05985915	0.04284621	0.08579668
HeLa shMITOL PolGA WT	97.6	111.2	121.6	111.7	134.1	136.5	0.87376902	0.82923192	0.89084249
HeLa shMITOL PEO #1	68.3	64	78.3	116.7	137.3	138.4	0.58526135	0.46613256	0.56575145
HeLa shMITOL PEO #2	73.9	69.9	84.5	99.6	133.1	123.3	0.74196787	0.52516905	0.68532036

Fig 5D

	Slot Blot				Southwestern			
	None	PolGA WT	PEO #1	PEO #2	None	PolGA WT	PEO #1	PEO #2
Expt #1	17.97	53.351	33.66	60.92	13.66	72.7	59.8	75.8
Expt #2	23.66	60.017	42.44	66.4	19.6	69.65	62.8	66.45
Expt #3	18.47	58.6	40.6	58.96	24	74.6	66.88	70.7

Fig 6C

	PolI WT			PEO #1			PEO #1 K1060R			PEO #2			PEO #2 K1060R		
Mitochondria	190.471	111.762	145.912	164.787	144.019	161.785	148.027	177.717	168.116	133.567	122.501	154.323	148.876	188.958	179.908
Post-trypsin	156.33	105.804	120.16	23.698	31.066	24.861	73.512	84.345	68.727	20.824	27.856	14.152	76.75	70.592	65.641
Mitoplast	134.912	92.283	114.838	24.867	21.385	15.185	67.524	72.747	74.62	19.136	19.517	15.187	72.087	79.777	73.094

Fig 6E

	Slot Blot					Southwestern				
	PolGA WT	PEO #1	PEO #1 K1060R	PEO #2	PEO #2 K1060R	PolGA WT	PEO #1	PEO #1 K1060R	PEO #2	PEO #2 K1060R
Expt #1	120.43	55.6	92.96	66.44	96.724	111.924	13.351	46.56	11.543	36.194
Expt #2	110.65	70.9	107.8	60.512	95.93	74.171	20.017	47.854	13.308	45.833
Expt #3	127.75	68.6	102.6	62.55	98.5	95.5	18.6	50.25	22.7	49.8

S1A Fig

Expt #1						
	Ct	ΔCt	ΔΔCt	2^-ΔΔCt		
**GAPDH**						
Vector	25					
Myc Mitol	24.89					
**Mitol**						
Vector	22.28	-2.72		1		
Myc Mitol	19.86	-5.03	-2.31	4.9588308		
**Pol GA**						
Vector	24.1	-0.9		1		
Myc Mitol	23.97	-0.92	-0.02	1.01395948		
Expt #2						
**GAPDH**						
Vector	28.56					
Myc Mitol	28.96					
**Mitol**						
Vector	26	-2.56		1		
Myc Mitol	24.18	-4.78	-2.22	4.65893435		
**Pol GA**						
Vector	30.87	2.31		1		
Myc Mitol	30.99	2.03	-0.28	1.21419488		
Expt #3						
**GAPDH**						
Vector	27.42					
Myc-Mitol	26.98					
**Mitol**						
Vector	30.22	2.8		1		
Myc-Mitol	27.96	0.98	-1.82	3.53081199		
**Pol GA**						
Vector	29.81	2.39		1		
Myc-Mitol	29.6	2.62	0.23	0.85263489		
Combined						
	Mitol (relative transcript levels)			PolGA (relative transcript levels)		
	Expt#1	Expt#2	Expt#3	Expt#1	Expt#2	Expt#3
Vector	1	1	1	1	1	1
Myc Mitol	4.958831	4.658934	3.5801	1.013959	1.214195	0.852635

S1B Fig

Expt #1						
	Ct	ΔCt	ΔΔCt	2^-ΔΔCt		
**GAPDH**						
siControl	24.42					
siMitol	25.55					
**Mitol**						
siControl	26.11	1.69		1		
siMitol	29.26	3.71	2.02	0.246558		
**PolyA**						
siControl	26.83	2.41		1		
siMitol	27.81	2.26	-0.15	1.109569		
Expt #2						
**GAPDH**						
siControl	25.2					
siMitol	25.66					
**Mitol**						
siControl	28.95	3.75		1		
siMitol	30.77	5.11	1.36	0.38958229		
**Pol Gamma A**						
siControl	28.04	2.84		1		
siMitol	28.53	2.87	0.03	0.979420298		
Expt #3						
**GAPDH**						
siControl	23.71					

siMitol	23.93					
**Mitol**						
siControl	30.02	6.31		1		
siMitol	31.76	7.83	1.52	0.348685917		
**Pol Gamma A**						
siControl	25.88	2.17		1		
siMitol	26.33	2.4	0.23	0.852634892		
Combined						
	Mitol (Transcript levels)			PolGA (transcript levels)		
	Expt#1	Expt#2	Expt#3	Expt#1	Expt#2	Expt#3
siControl	1	1	1	1	1	1
siMitol	0.246558	0.389582	0.348686	1.109569	0.97942	0.852635

S4C Fig

	PolGA			hsp90			PolGA/hsp90		
	Expt #1	Expt #2	Expt #3	Expt #1	Expt #2	Expt #3	Expt #1	Expt #2	Expt #3
Untransfected	68.3	55.8	72.4	146.5	113.9	132.7	0.4662116	0.48990342	0.54559156
PolGA WT	148.6	142.7	145.9	149.8	146.6	146.3	0.99198932	0.973397	0.99726589
PEO #1	162.5	157.1	172	172.9	155.7	162.3	0.93984962	1.00899165	1.05976587
PEO #2	150	152.4	159.8	166.1	159.2	157.3	0.90307044	0.95728643	1.0158932
PEO #3	164.3	176.3	166	152.7	156.6	152.1	1.07596595	1.12579821	1.09138725
PEO #4	160	154.3	158.5	149.8	149.3	151.3	1.06809079	1.03348962	1.04758757

S5C Fig

	10 nM			20 nM		
	Expt #1	Expt #2	Expt #3	Expt #1	Expt #2	Expt #3
POLGA WT	70.312	80.512	83.174	118.566	116.832	120.661
F961S	17.844	15.178	7.677	25.461	21.372	17.074
A467T	7.321	6.614	5.91	11.197	16.278	13.815
W748S	20.809	34.729	21.179	40.855	51.481	26.543
Y955C	8.059	6.557	5.398	21.473	16.739	9.812

S5E Fig

	10 nM			20 nM		
	Expt #1	Expt #2	Expt #3	Expt #1	Expt #2	Expt #3
POLGA WT	94.503	77.848	89.352	97.849	88.704	77.187
F961S	9.617	3.212	0.812	13.805	6.046	3.623
A467T	5.02	2.843	2.808	3.077	4.687	4.827
W748S	1.01	7.159	5.379	4.244	10.205	8.601
Y955C	83.155	90.478	84.977	86.148	95.14	72.129

S6B Fig

	Signal Intensity			Adjusted Signal Intensity			Adjusted Signal Intensity/100		
	Expt #1	Expt #2	Expt #3	Expt #1	Expt #2	Expt #3	Expt #1	Expt #2	Expt #3
PolGA WT	143.1	102.53	115.5	100	100	100	1	1	1
PEO #1	61.533	37.9361	58.905	43	37.0000002	51.0000002	0.43	0.37	0.51

PEO #2	22.896	21.5313	35.805	16	21.0000001	31.0000002	0.16	0.21	0.31

S6D Fig

	PolGA (Soluble)			PolGA (Insoluble)		
	Expt #1	Expt #2	Expt #3	Expt #1	Expt #2	Expt #3
PolGA WT	46.4	54.8	59.4	86.5	93.8	79.6
PEO #1	47.5	44.6	42.4	156.3	165.8	166
PEO #2	44.5	45	48	150.6	153.9	165.3
PEO #3	46.5	42	50	76.5	68.1	69.6
PEO #4	47.6	50.1	49.2	90.3	50.29	78.3

S6F Fig

	PolGA (Soluble)			PolGA (Insoluble)		
	Expt #1	Expt #2	Expt #3	Expt #1	Expt #2	Expt #3
PEO #1	130.4	119.3	112.8	169.7	157.2	162.6
PEO #1 K1060R	128.4	132.6	106.8	30.4	43.2	48.7
PEO #2	130.5	122	128	158.5	170.2	169.4
PEO #2 K1060R	146.4	132.1	138.1	35.6	40.9	40.2
