# Supplementary material for: MITOL-dependent ubiquitylation negatively regulates the entry of PolγA into mitochondria
Source: PLoS Biol. 2021 Mar 3;19(3):e3001139. doi: 10.1371/journal.pbio.3001139 (PMC7959396; doi:10.1371/journal.pbio.3001139)

Figure 1

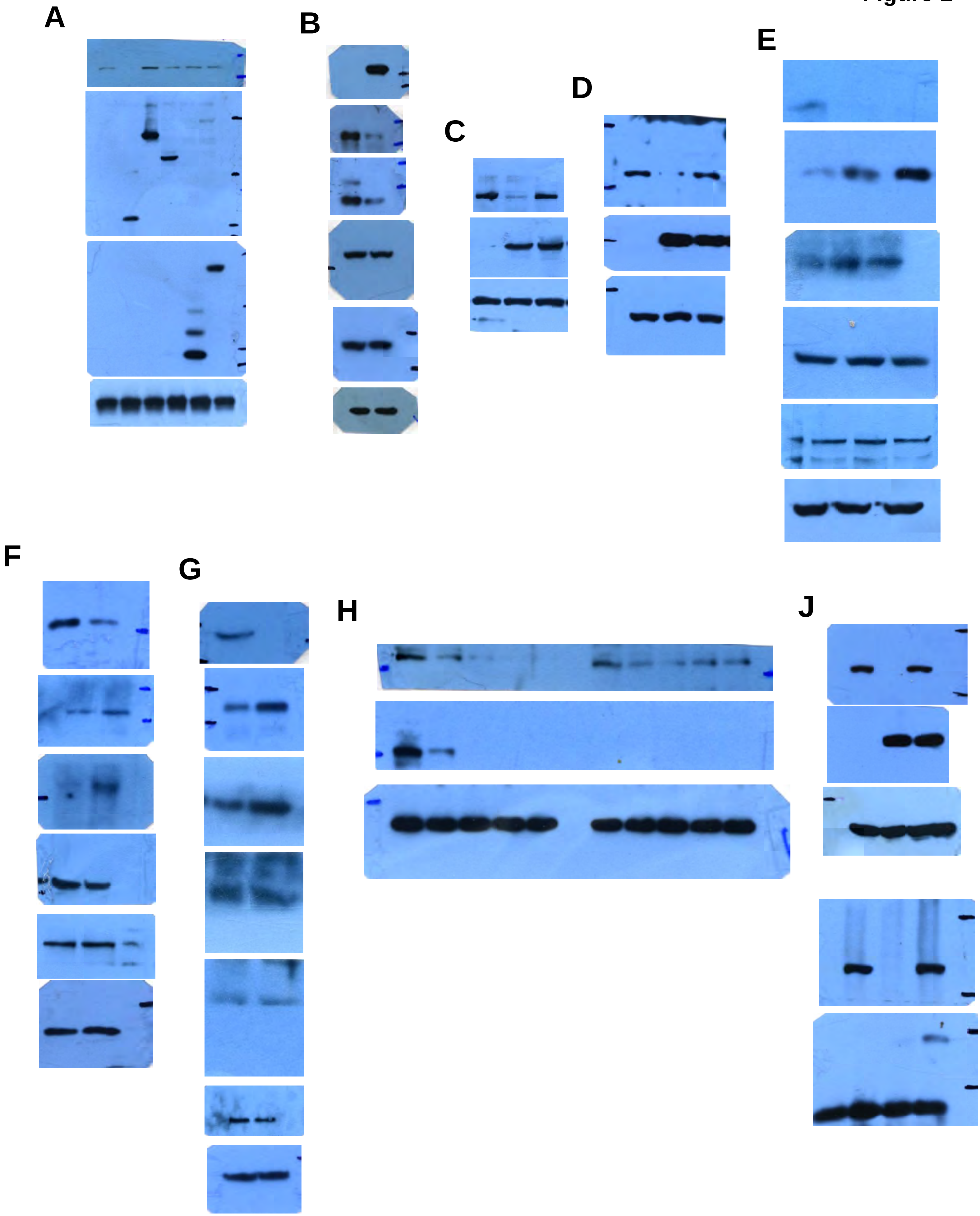

Figure 2

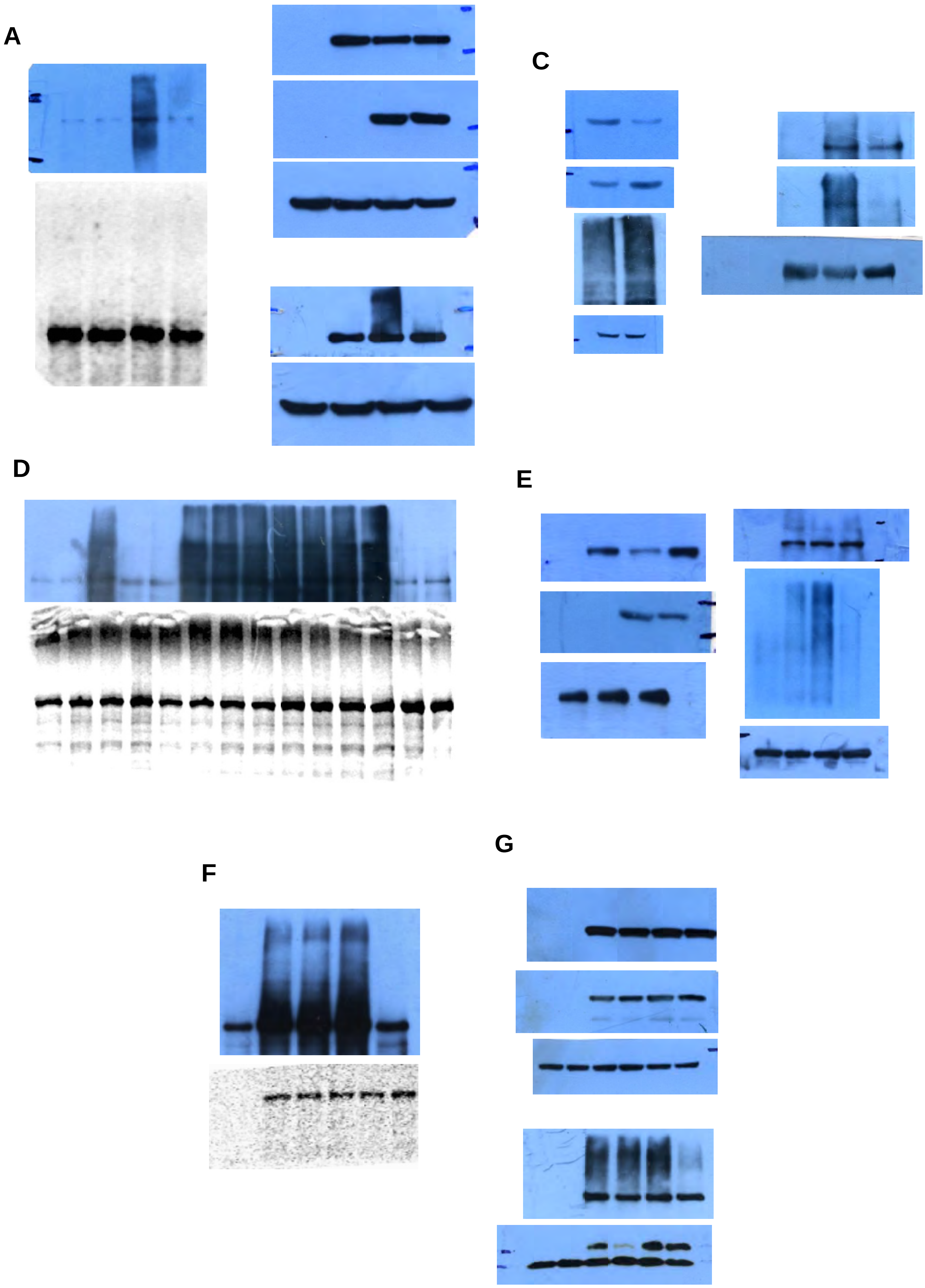

Figure 3

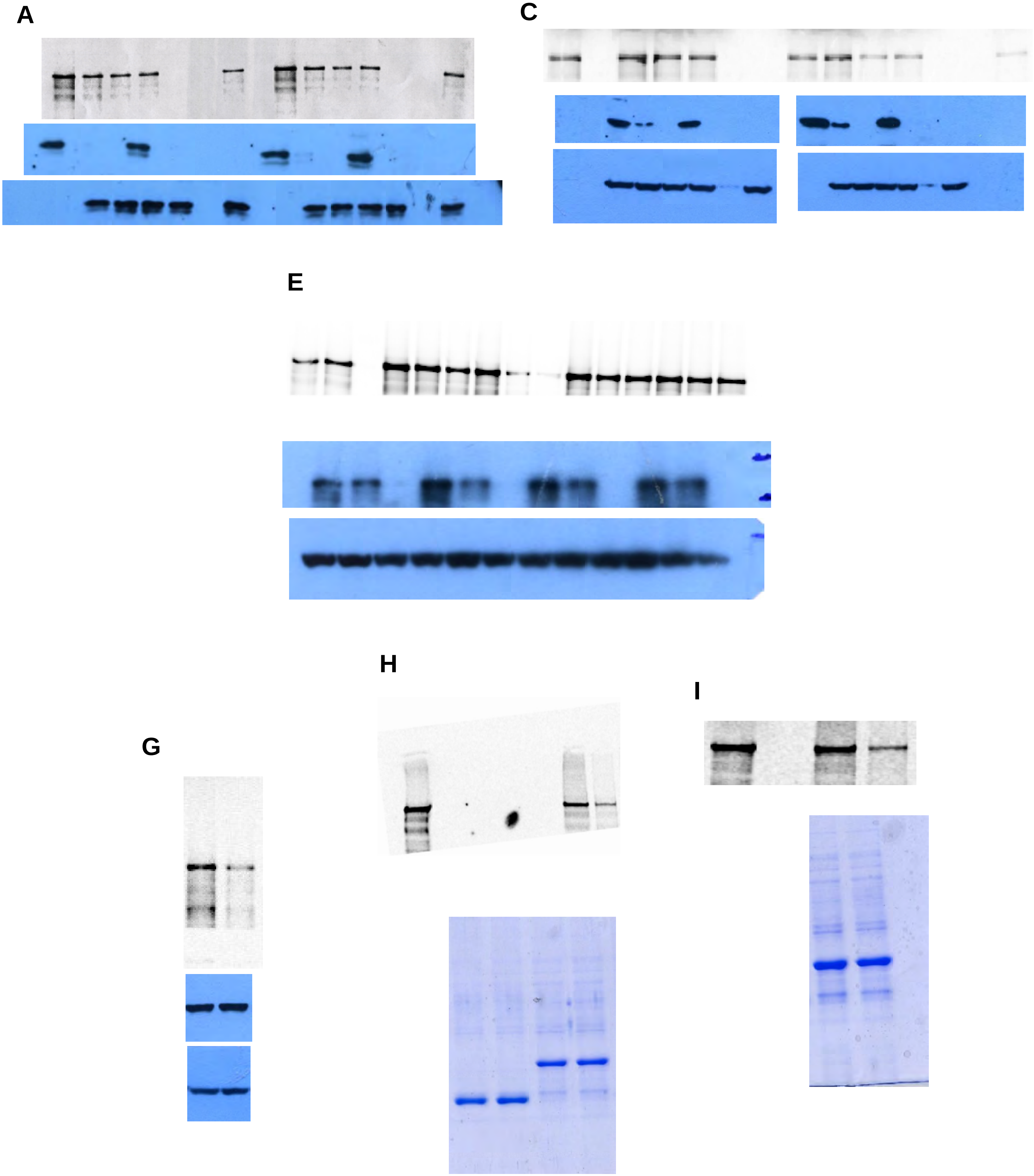

Figure 4

A

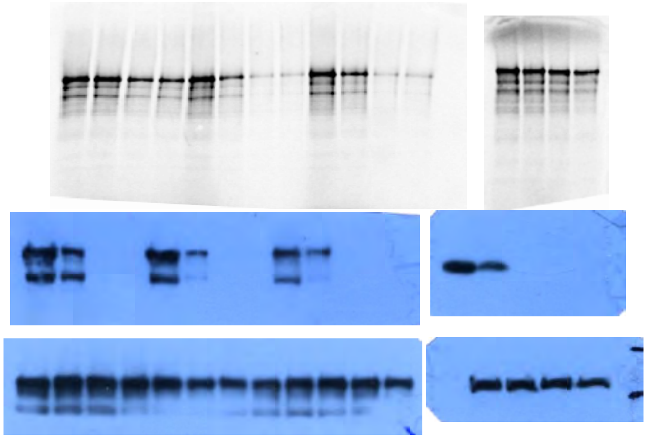

C

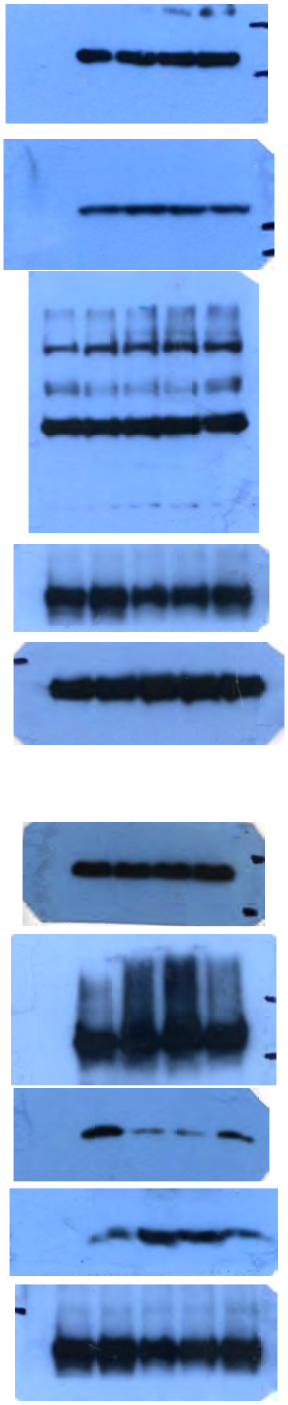

D

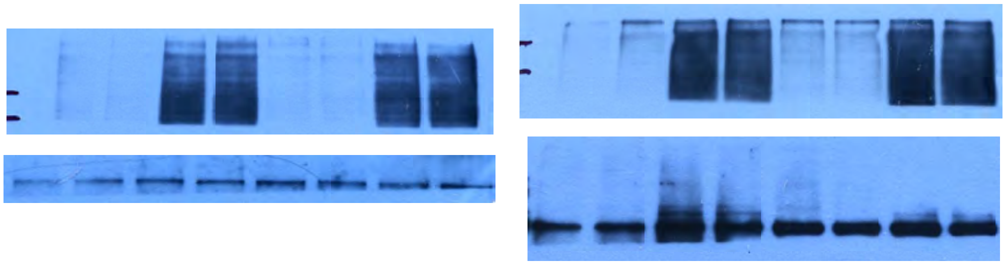

E

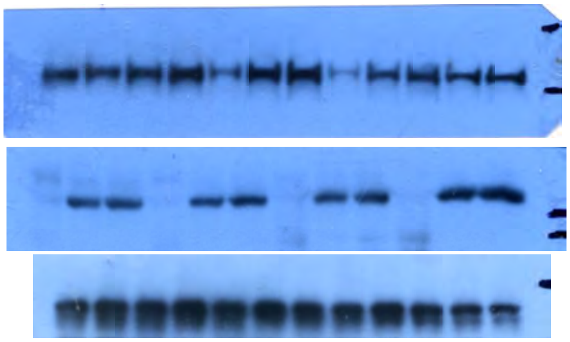

F

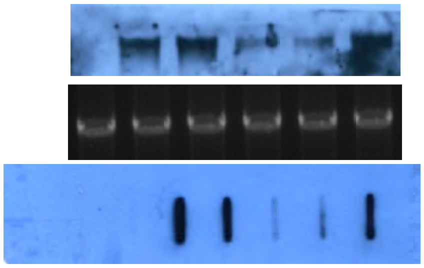

Figure 5

A

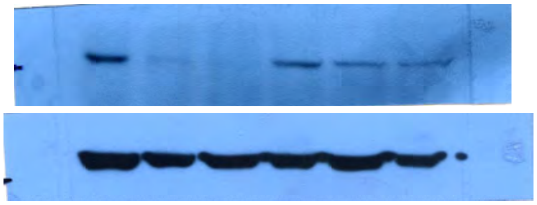

B

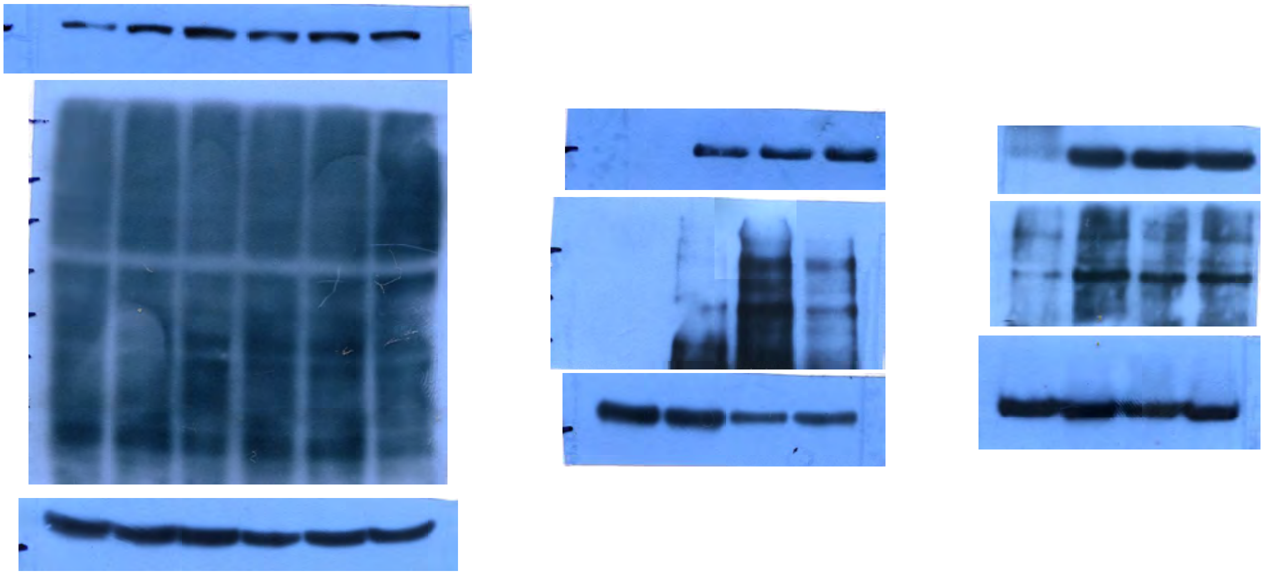

C

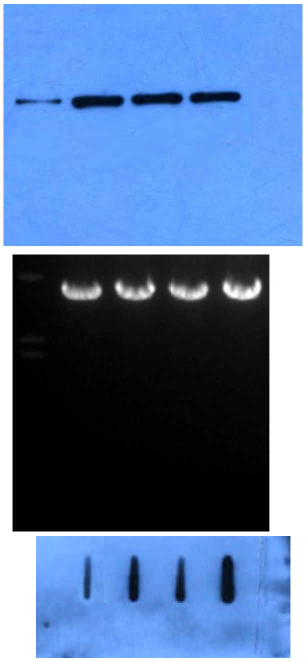

Figure 6

A

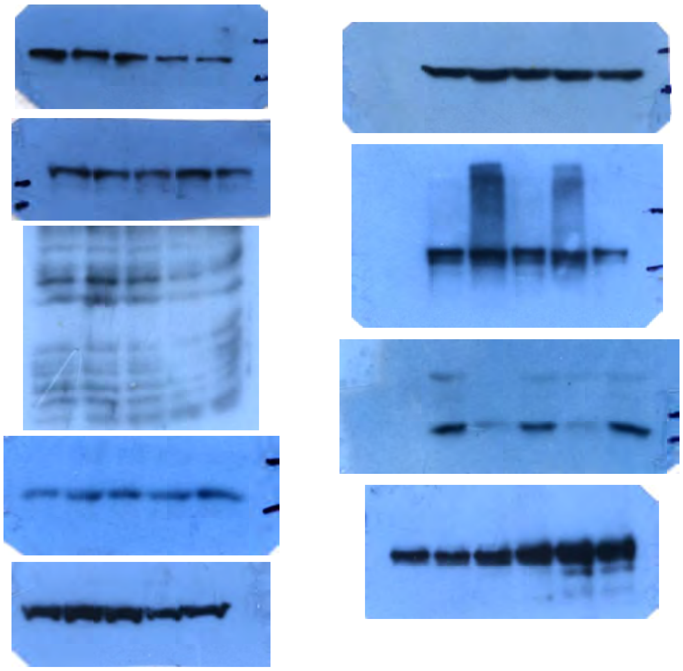

B

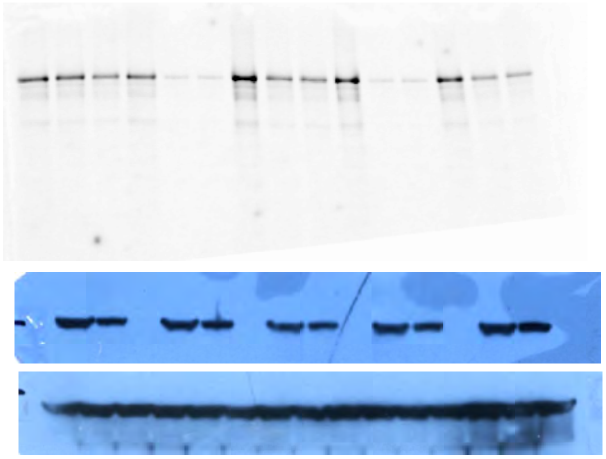

D

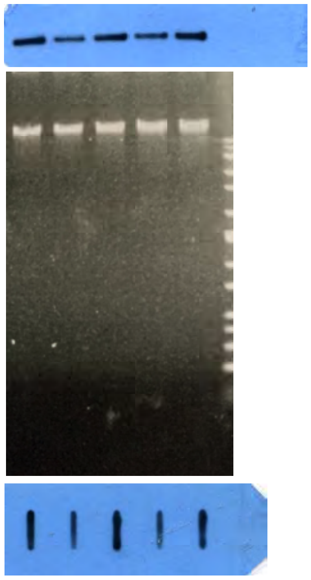

Figure S1

A

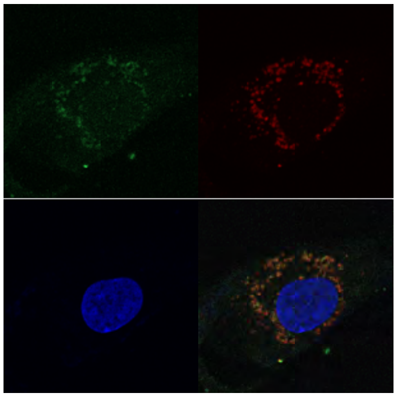

D

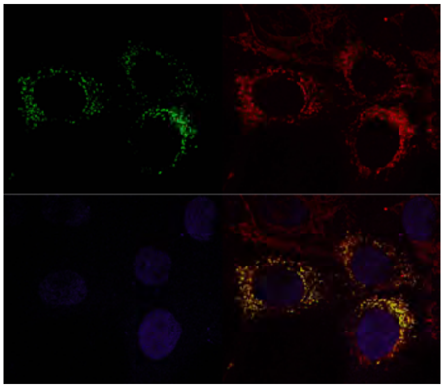

E

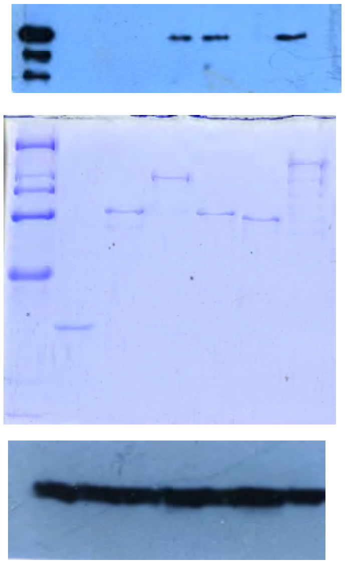

F

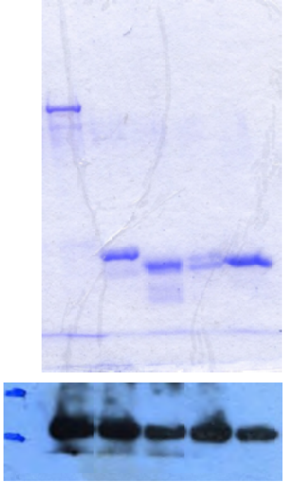

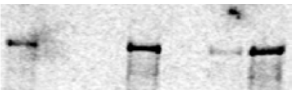

Figure S2

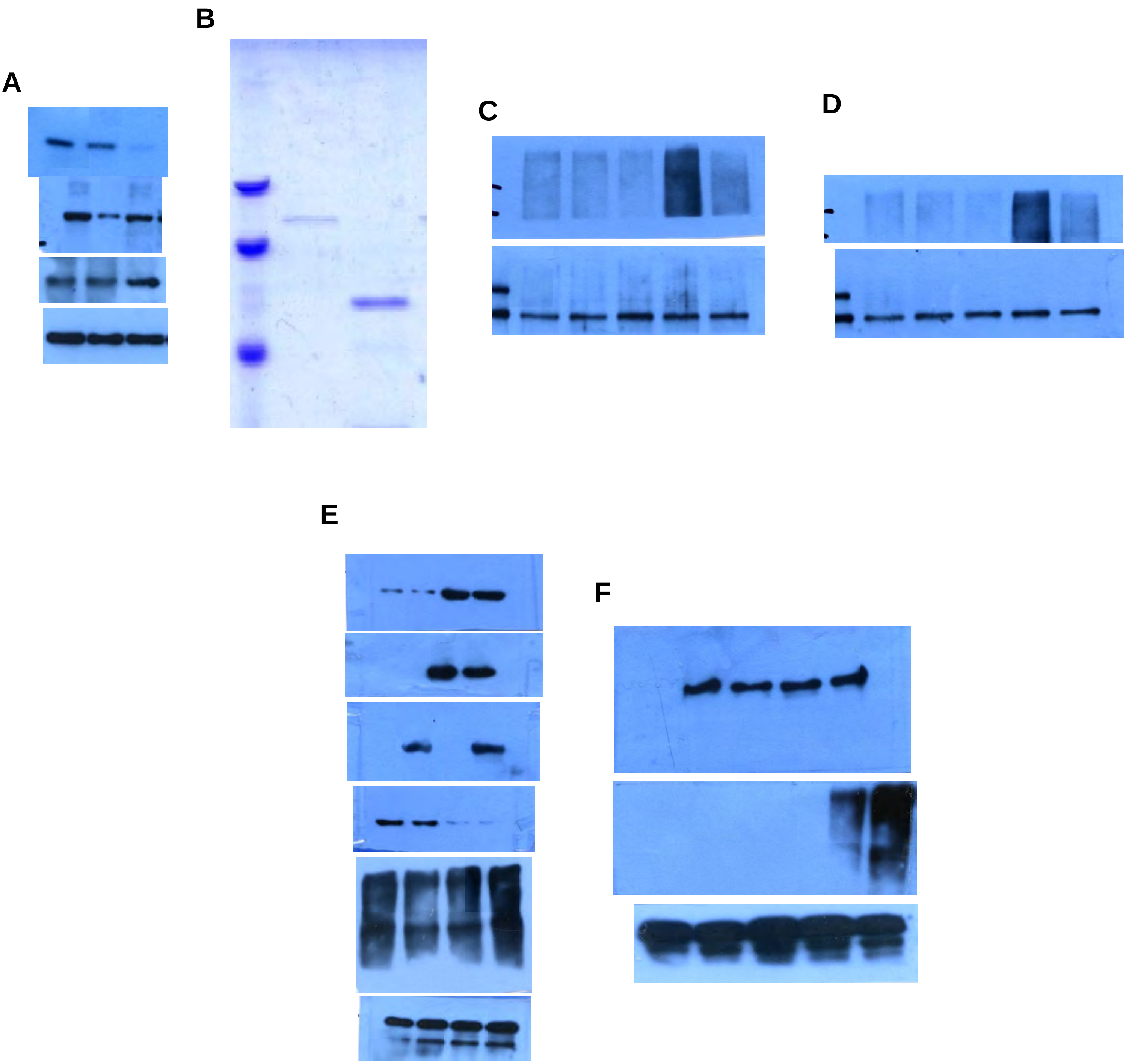

Figure S3

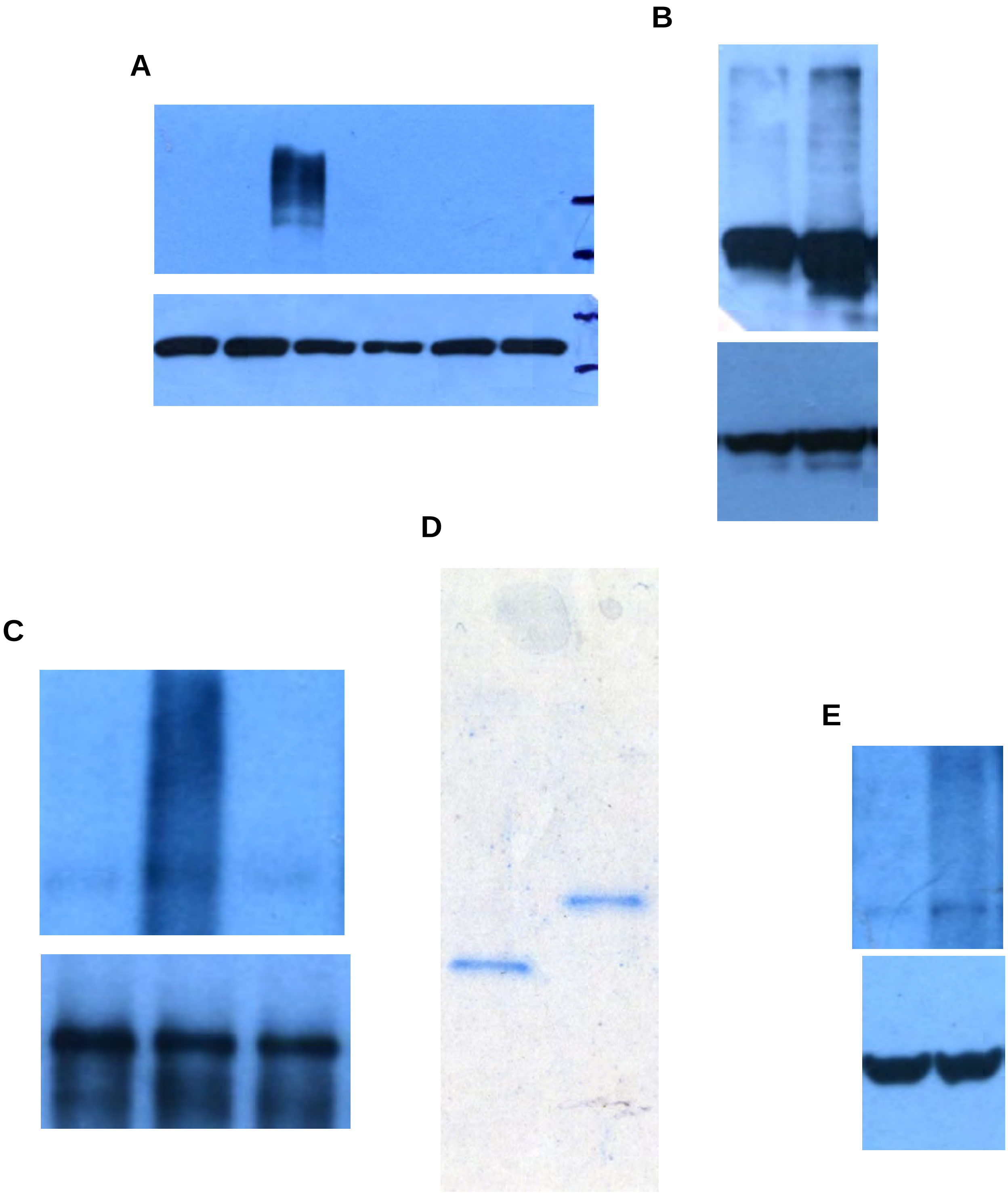

Figure S4

A

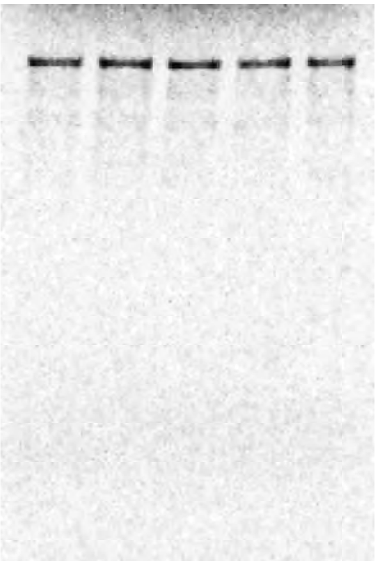

B

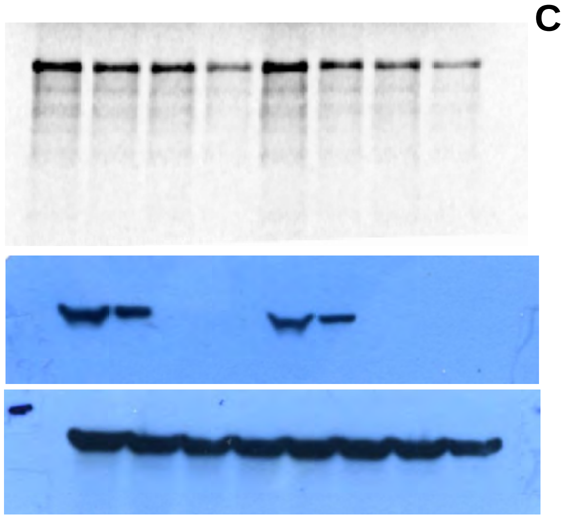

C

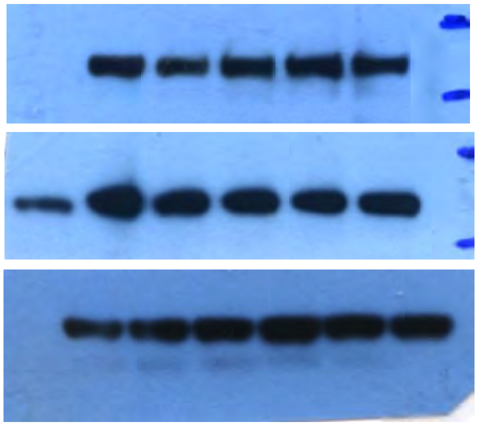

D

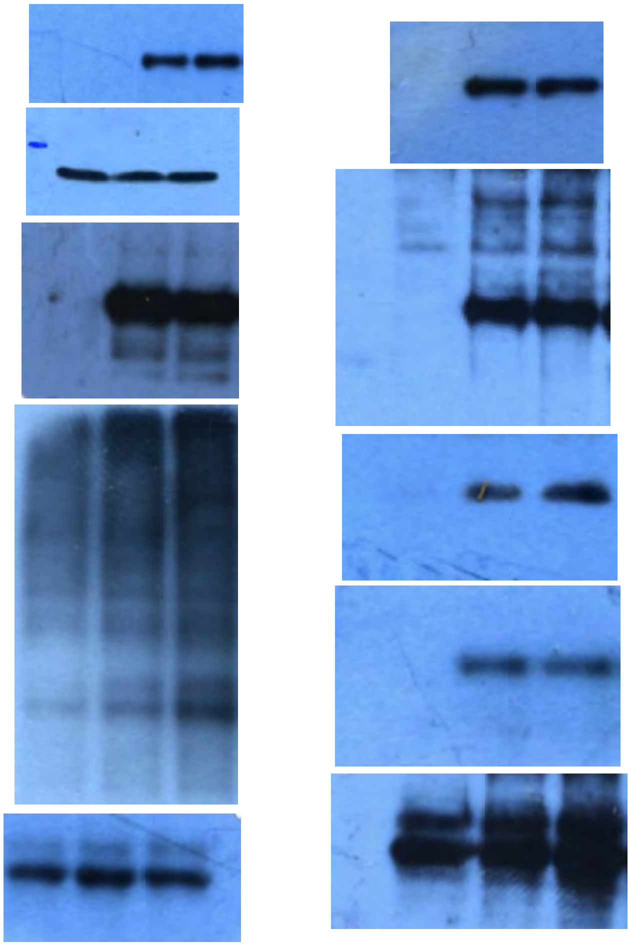

Figure S5

A

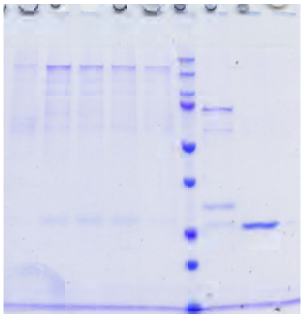

B

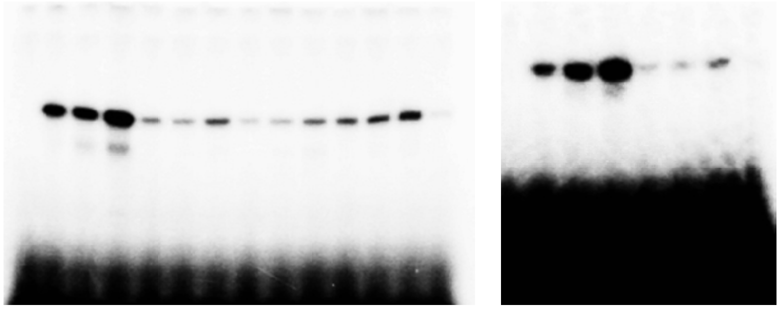

D

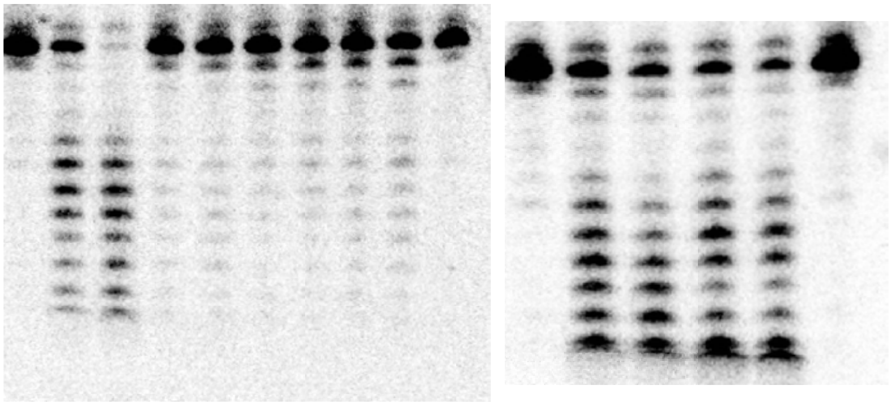

Figure S6

C

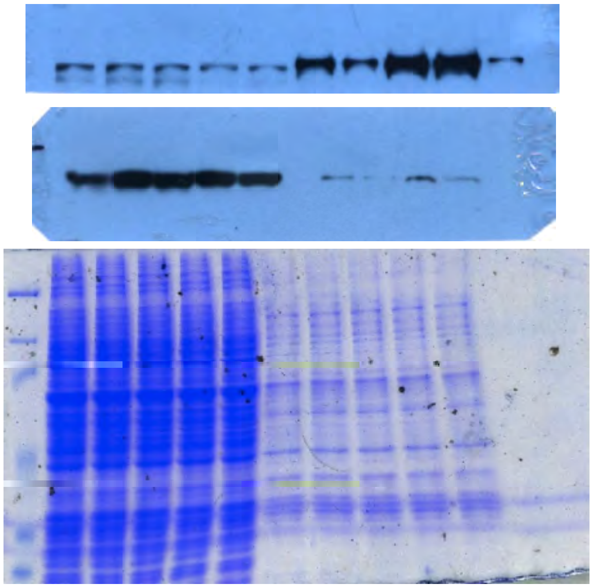

A

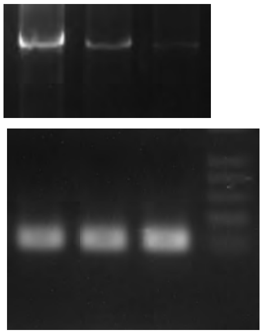

E

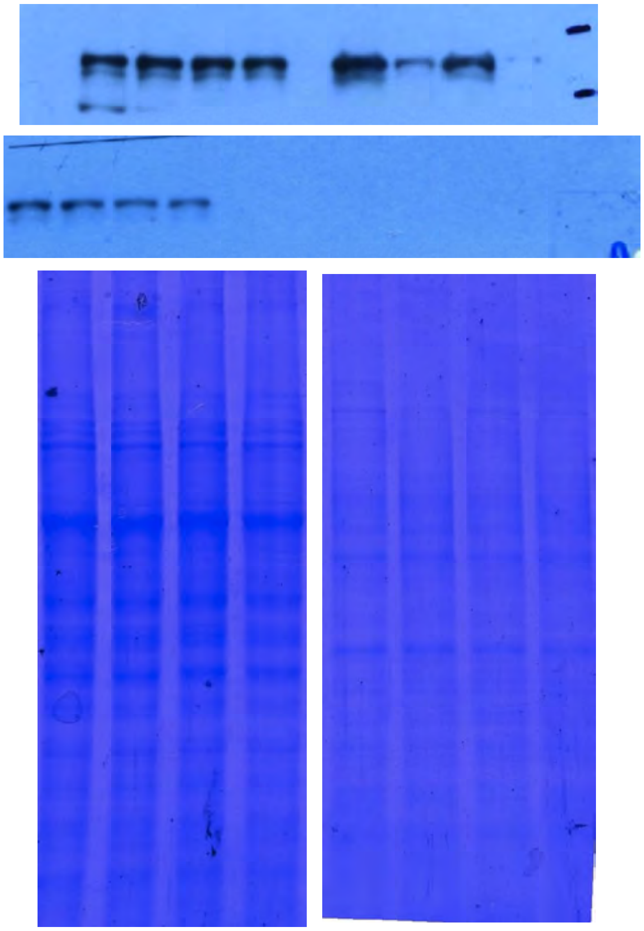

Supplement: S1 Original Images — (PDF) [file pbio.3001139.s014.pdf]
