# Supplementary material for: MITOL-dependent ubiquitylation negatively regulates the entry of PolγA into mitochondria
Source: PLoS Biol. 2021 Mar 3;19(3):e3001139. doi: 10.1371/journal.pbio.3001139 (PMC7959396; doi:10.1371/journal.pbio.3001139)
Supplement: Attachment — Submitted filename: Response to reviewers comments.pdf [file pbio.3001139.s017.pdf]

Response to reviewer's comments

Reviewer #1:

****Summary****

In this manuscript that authors have dissected how a key mitochondrial replication protein PolγA is ubiquitylated on the mitochondrial outer membrane by MITOL, an E3 ligase present on the mitochondrial outer membrane. Using a number of in vivo and in vitro assays the authors demonstrate that MITOL ubiquitylates PolγA at specific residues using K6 linkage. Only non-ubiquitylated or hypo-ubiquitylated PolγA can enter mitochondrial matrix and this enhances their functions during mtDNA replication. Further mutant PolγA variants present in some of the PEO patients are hyper-ubiquitylated by MITOL. This results in formation of "aggregate like structures" for mutant PolγA which cannot enter mitochondria and hence is less effective during mtDNA replication. Finally two independent lines of evidence show that non-functional PolγA patient mutants can be reactivated. The results are compelling and the relevant experimental controls are present. However certain additional explanations are needed and some experiments need to be performed to further strengthen the manuscript.

Response:

We thank the reviewer for the positive comments.

****Major Points****

Major query #1

Overall the concept is that the mere presence of mitochondrial localization signal is not sufficient for proteins to enter into the mitochondrial matrix and finer regulatory control like ubiquitylation is necessary. This should be more elaborated in the Discussion.

Response:

We completely agree with the reviewer. We have included this pertinent point in the Discussion in the revised version of the manuscript (page 14).

Major query #2

Figure 2 and others: Ubiquitylation of PolγA were observed by ladder formation with anti-PolγA antibody, but it seems that the amounts of substrate (PolγA) were not equal. It looks like WB data probed with anti-ubiquitin antibody. In contrast, the same amounts of 35S PolγA are almost equal. The authors should explain this difference and the reason why no ladder formation of 35S PolγA was observed.

Response:

We would like to point it out that all the blots for *in vitro* ubiquitylation were carried out with mostly with anti-PolγA antibody and in a few cases with anti-ubiquitin antibody. The amount of substrate taken was always equal as determined either by the 35^S PolγA autoradiogram or

anti-PolγA western blot analysis. Infact every time after the westerns were, done the same blot was exposed to get the 35^{S} PolγA autoradiogram.

Further, we did note that PolγA ubiquitylated smears were not observed upon autoradiography. This was due to the fact that the SDS-PAGE gels had to be subjected to fluorography (as S^{35} Methionine is a weak beta emitter), the gels dried at 65°C for 3-4 hrs, before being exposed for autoradiography. The process of fluorography and/or the heating the gels during the drying process degraded the ubiquitylated products for a high molecular weight substrate like PolγA. This explanation has been provided in the Materials and Methods section of the revised version of the manuscript (page 18).

Major query #3

Figure 2C: The authors showed that, in vitro ubiquitination assay, MITOL ubiquitinates PolγA in a K6-specific manner. In my experience, in vitro, E3 ligases randomly add ubiquitin chains on substrates. It is speculated that E2 and other factors may be involved in the determining of pattern of ubiquitin chain formation in cells. Is this K6-specific Ub in vitro due to the specific property of PolγA? This should be more elaborated in the Discussion.

Response:

We thank the reviewer for this query.

We believe that E3s confer specificity to the ubiquitylation reactions. (For reviews on this subject please consider PMID: 19489725, 26862208)

In the present case we firmly believe that the K6-specific ubiquitylation on PolγA by MITOL is a specific property of PolγA. This based on *in vitro* ubiquitylation data in Figure 2D and *in vivo* western analysis with very specific K6-affimer data in Figure 2E.

For *in vitro* determination of specificity, we have used two types of ubiquitin mutants: “R” mutants and “O” mutants. In “R” ubiquitin mutants, only a particular lysine residue in ubiquitin is mutated while in “O” ubiquitin mutants only one lysine is present on ubiquitin while all other six lysine residues on ubiquitin are mutated to alanine residues. This information has been provided in both the Results and the Materials and Methods section of the revised version of the manuscript (pages 8 and 18). Usage of these ubiquitin mutants show that PolγA is ubiquitylated by MITOL via K6-linkage. For *in vivo* validation we have used the K6-affimer – which again validate the K6-linkage on PolγA. We have also elaborated this in the Discussion (Page 15).

Major query #4

Figure 4E: using Southwestern and slot blot analysis authors show that some of the PEO mutants do not incorporate BrDU and hence they are presumably defective in mtDNA replication. Will the same PEO mutants behave similarly when used for in vitro exonuclease and polymerase assays?

Response:

We thank the reviewer for the comments.

We have carried out both *in vitro* exonuclease and polymerase assays with wildtype PolγA and the four tested PEO mutants. This data has been incorporated in the revised of the manuscript (Figure S5).

Major query #5

How does non-ubiquitylated PolγA which enters into the mitochondrial matrix undergo turnover? Are these proteins again exported out to the mitochondrial surfaces to be targeted by MITOL? Or do these proteins undergo turnover inside mitochondrial matrix?

Response:

We thank the reviewer for the very interesting question. It is possible that PolγA which enters into the mitochondria can undergo turnover in two ways: either exported out again of the mitochondria (as postulated for other proteins PMIDs: 14731958, 11251115, 9482871) or becoming a target of mitochondrial proteases (as reviewed in PMID: 28589125, 32075415). While this question is very interesting, it falls outside the purview of this manuscript.

Major query #6

Two of the four PEO mutants do not form aggregates, are not degraded by MITOL and enter into the mitochondria with equal efficiency as wildtype PolγA. What is the reason of discrimination between the PEO mutants which allows some mutants but not others to be a MITOL target?

Response:

We thank the reviewer for this very interesting scientific comment.

In this case we are dealing with single point mutations of PolγA which cause drastic changes in disease causing phenotype – one class of PolγA mutants (PEO mutants #1 and #2) are predominantly present in the insoluble fraction and remain outside the mitochondria. The second class of PolγA mutants (PEO mutants #3 and #4) go inside the mitochondria, like wildtype PolγA.

We hypothesized that this occurs due to the different extents of MITOL dependent ubiquitylation on PolγA variants (Discussion, Page 14). To test this hypothesis GST tagged wildtype PolγA and the four PEO mutants were purified in bound form. We wanted to carry out MITOL-dependent *in vitro* ubiquitylation on Glutathione Sepharose bound PEO mutants. Post-ubiquitylation we hoped to elute out the ubiquitylated products using reduced glutathione (GSH) and then carry out tryptophan fluorescence experiments to understand the alterations in the conformation in the PolγA variants upon ubiquitylation. Unfortunately in our hands the *in vitro* ubiquitylation by MITOL on bead bound GST-PolγA and PEO mutants did not work. We hope to continue this aspect of work (using His tagged PolγA variants), but for the present manuscript we could not answer this query.

Minor Points

Minor query #1

Figure 4: They showed the ubiquitination and proteasomal degradation of PEO mutants by MITOL overexpression. It is better to show accumulations and decreased mitochondrial import of PEO mutants in MITOL-knockdown cells. If possible, CHX assay should be performed.

Response:

To answer this query we have used isogenic HeLa cells (HeLa shGFP and HeLa shMITOL). In these cells we overexpressed equal amount of Flag tagged WT and PEO mutants, isolated the mitoplast and checked the levels of imported PolγA proteins into the organelle. Ablation of MITOL in HeLa shMITOL cells led to the entry of both PEO mutants #1 and #2 into mitoplast to almost equal extent as wildtype PolγA (Figure 5A).

We believe than we have provided evidence that PEO mutants #1 and #2 cannot enter into mitochondria due to mechanistic defect in mitochondrial import. Hence we feel that CHX experiment will not be informative in MITOL deficient background as this experiment measures the half-life of the proteins and not the extent of mitochondrial import. Hence CHX experiment for PEO mutants #1 and #2 was not attempted.

Minor query #2

The authors should speculate the fate of the cells which accumulate the PEO patient mutants. If possible, a live cell experiment should be performed to know whether the cells showing accumulation of these mutant proteins survive in subsequent generations.

Response:

We appreciate the reviewer's comments. This line of study is part of an active research project in the lab whereby stable lines expressing PolγA wildtype and the four mutants are in the process of being generated. These stable lines will be characterized using live cell microscopy. However this component will be outside the purview of the present manuscript.

Minor query #3

It is very interesting to see if USP30 suppresses PolγA ubiquitination by MITOL. If possible, please check it.

Response:

We appreciate the reviewer's query. We have used both *in vitro* and *in vivo* assays to determine whether USP30 suppresses PolγA ubiquitination by MITOL.

For this purpose we standardized a modified ubiquitylation cum deubiquitylation assay where carried out *in vitro* ubiquitylation of PolγA by MITOL in presence or absence of recombinant USP30 under two conditions – (a) recombinant USP30 was added simultaneously i.e. during MITOL dependent PolγA ubiquitylation or (b) recombinant USP30 was added after MITOL

dependent PolγA ubiquitylation for another 90 minutes. In both cases USP30 could deubiquitylate MITOL mediated ubiquitylation on PolγA (Figure S2C, S2D).

However *in vivo*, when we overexpressed USP30 in presence of MITOL, the decrease in the protein levels of PolγA by MITOL could not be reversed (Figure S2E). Similarly expression of USP30 could not decrease the extent of MITOL-dependent ubiquitylation on PolγA (Figure S2F). Hence, we believe that *in vivo* USP30 may act as a deubiquitylase to MITOL ubiquitylated PolγA only under very specific physiological conditions, which needs to be further investigated.

Reviewer #1 (Significance):

****Nature and significance of the advance.****

This work reveals novel mechanisms in mitochondrial DNA replication and quality control, and suggests the molecular pathology of PEO.

****Compare to existing published knowledge.****

This paper basically follows the concept of a previous paper that "Dynamic Regulation of Mitochondrial Import by the Ubiquitin System" published in Mol Cell 2020, and shows further development in the regulation of mitochondrial import system.

****Audience.****

Relatively broad

****My expertise.****

Mitochondrial biology, especially ubiquitin-mediated signaling on the mitochondrial outer membrane

Response:

We thank the reviewer for the positive comments.

Reviewer #2 (Evidence, reproducibility and clarity:

This is an interesting manuscript by Hussain et al. who proposes that mitochondrial-disease associated mutations in PolgA lead to defects in their mitochondrial import. Specifically, mitochondrial-surface attached ligase MITOL promotes ubiquitination of PolgA, impairing its import. The "non-ubiquitinatable" PolgA are imported more efficiently, leading to improved PolgA function. The manuscript builds upon the previous data on quality control pathways at the mitochondrial translocon and suggests engagement of these pathways on mutated mitochondrial proteins limit their import and function. Remarkably, mutating the lysine that is ubiquitinated in mutant PolgA restores its import and function. The biochemical data presented extensively examines the ubiquitination of PolgA by MITOL. Additional experiments providing technical controls and linking the impact of ubiquitination of mutants to March5 and mitochondrial function should further improve the manuscript.

The studies shown could be improved by providing the quantification of immunoblotting experiments as suggested below. The additional experiments suggested are mainly aimed at showing that endogenous MITOL in cells carry out the functions as hypothesized by the authors.

Response:

We thank the reviewer for the positive comments.

Major query #1

The authors should also improve statistical comparisons by using ANOVA and post-hoc tests (in multiple comparisons) rather than using multiple t-tests.

Response:

We appreciate the reviewer's query. We have re-done the statistical analysis of all the quantifications. Almost in all cases either One-way ANOVA or Two-way ANOVA have been used. The statistical analysis performed in each study has been listed in Table S6. A section has been added in the Methods section under the heading "statistical analysis" (page 23) – where we have indicated that all data have been presented as mean ± SD. * $p < 0.05$, ** $p < 0.01$, *** $p < 0.001$, **** $p < 0.001$.

Major query #2

Does mRNA levels of PolgA change upon MITOL over expression or knockdown?

Response:

We have carried out the suggested experiment. We have either overexpressed exogenous MITOL or ablated endogenous MITOL and subsequently checked the mRNA levels of PolγA. Neither the overexpression nor the ablation of MITOL had any affect on PolγA transcript levels (Figure S1A. S1B).

Major query #3

In experiments where immunoblotting was performed >3 times, please show quantifications in a bar plot especially in experiments where the change in immunoblot signal is small e.g. 1C, control vs MG132; 2D, control vs MITOL etc.

Response:

We have positively responded to the suggestion of the reviewer. Hence we have provided quantification in a bar plot for a number of experiments involving immunoblots. Further we have done the statistical analysis of all the quantifications. The statistical analysis in each case has been listed in Table S6.

Major query #4

Please provide additional markers in Fig. 1E-G to show overall mitochondrial proteome is intact. For example, show levels of proteins studied in Fig 1B in these MITOL-depletion experiments.

Response:

We appreciate the reviewer's query. We have provide additional markers in Figure 1E, 1F, 1G to show that the mitochondrial proteome is intact when MITOL is depleted. This data has been incorporated in the revised version of the manuscript.

Major query #5

Why does the "non-ubiquitinatable" K1060R mutant not stabilized (increased protein levels) compared to other mutants in Fig 2G?

Response:

We appreciate the reviewer's concern. This is to do with the experimental conditions i.e. the time-point when the lysates were made. The time point used for this experiment (24 hrs post-transfection) was used to detect robust ubiquitylation (or its loss) (as seen in the IP/Western blot) (Figure 6A).

Major query #6

Does knockdown of MITOL eliminate ubiquitination of PolgA?

Response:

We have addressed the reviewer's query by using asynchronously grown HeLa shGFP and HeLa shMITOL cells. PolγA was immunoprecipitated from both cell types and checked for its relative ubiquitylation in the two cell types. Indeed knockdown of MITOL eliminated ubiquitination of PolγA (Figure 2C).

Major query #7

Does ubiquitination additionally alter interaction with TOM70 receptor (a receptor that is responsible for importing aggregation-protein/hydrophobic substrates)?

Response:

While we appreciate the reviewer's question – we think that this question is outside the purview of this manuscript.

Major query #8

What are the consequences of K6-mediated ubiquitination on PolgA? Are the effects described in Fig1 due to K6-ubiquitination? Does inhibition of proteasomes stabilize the K6-linked PolgA substrate?

Response:

Again we appreciate the reviewer's question. However repeating the entire set of experiments in Figure 1 with specific emphasis on K6-ubiquitylation was not possible. However it is to be noted that we have done the following key linking experiment to show that (a) ubiquitylation of PolγA *in vitro* is via K6-linkage using all the available ubiquitin mutants (Figure 2D); (b) ubiquitylation of PolγA *in vivo* is via K6-linkage using the K6-affimer (Figure 2E); (c) ubiquitylation of the PEO mutants #1, #2 *in vitro* is also via K6-linkage using K6O ubiquitin mutant i.e. only via linkage (Figure 4D).

Major query #9A

Did the authors use MITOL-ubiquitinated substrate in the import reactions shown in Fig 3? This is suggested in the methods but not clear in the main text.

Response:

Yes – we had used ubiquitylated PolγA. We have now added this clarification in the main text of the Result section (page 9) and also in the figure legend of Figure 3A (page 36).

Major query #9B

If substrates that were in vitro ubiquitinated were used for import reactions, please show the efficiency of ubiquitination on Western blots.

Response:

We have provided multiple western blots showing that PolγA was ubiquitylated by MITOL (please see Figure S3A, S3B, S3C and S3E).

Major query #9C

What fraction of presented substrate is ubiquitinated in Fig 3C?

Response:

A significant amount of the substrate is ubiquitylated – as will be apparent from the western blots in Figure S3A, S3B, S3C and S3E. However it is difficult to quantitate the exact percentage (or fraction) of ubiquitylation without quantitative mass spec analysis. We think this line of experimentation is outside the purview of this manuscript.

Major query #9D

If PolgA substrate is ubiquitinated, why does it run at the same molecular weight as non-Ub? Input for the Ub PolgA should also be shown in this panel.

Response:

It should be noted that all import assays were assayed by autoradiography. It is obvious that PolγA ubiquitylated smears were not observed upon autoradiography. This was due to the fact that the SDS-PAGE gels had to be subjected to fluorography (as S^{35} Methionine is a weak beta emitter), the gels dried at 65°C for 3-4 hrs, before being exposed for autoradiography. The process of fluorography and/or the heating the gels during the drying process degraded the ubiquitylated products for a high molecular weight substrate like PolγA. This explanation has been provided in the Materials and Methods section of the revised version of the manuscript (page 18).

In Figure 3A, the experiment was done using ubiquitylated PolγA. The Input given in Figure 3A is for ubiquitylated PolγA.

Major query #10

Are the PEO patient 1 and 2 PolgA Ub-ed by K6 linkage?

Response:

We thank the reviewer for asking this interesting and pertinent query. We have carried out *in vitro* ubiquitylation assays for PolγA wildtype and the two PEO mutants (mutant #1 and 2). In these assays we have used both wildtype ubiquitin and K6O ubiquitin mutant. Robust ubiquitylation of PEO mutants #1 and #2 when using K6O mutant (where only the lysine at 6th position on ubiquitin is present and all other lysines are mutated to alanines), indicated that like wildtype PolγA these two PEO mutants are also ubiquitylated via K6-linkage (Figure 4D).

Major query #11

Does knockdown of MITOL reduce ubiquitination and promote mitochondrial import of mutant PEO patient 1 and 2 PolgA in cells?

Response:

We appreciate the reviewer's query. To answer this query we have used HeLa shMITOL and HeLa shGFP cells. In these cells we expressed equal amounts of Flag tagged WT and PEO mutants #1 and #2. We saw that the ablation of MITOL in HeLa shMITOL cells led to the entry of both PEO mutants #1 and #2 into mitoplast, to equivalent levels as PolγA WT (Figure 5A).

Next, we wanted to determine the extent of ubiquitylation of these two mutants in comparison to PolγA WT in mitochondrial lysates (Figure 5B, left). As expected the extent of ubiquitylation in PEO mutant #1 and #2 was much higher than PolγA WT in HeLa shGFP cells (Figure 5B, middle). However, in HeLa shMITOL cells low but equivalent levels of ubiquitylation were observed for all the three PolγA variants (Figure 5B, right) indicating that the lack of MITOL was the critical factor for their successful mitochondrial entry into the mitochondria.

Major query #12

Does MITOL knockdown rescue the BrdU incorporation defects?

Response:

We appreciate the reviewer's question. We hypothesized that if ubiquitylation by MITOL is preventing PEO mutants #1 and #2 from entering mitochondria, ablation of MITOL in HeLa shMITOL cells should allow their entry and Brdu incorporation. Both Southwestern and slot blot analysis indicated that HeLa shMITOL cells expressing PolγA WT or PEO mutant #1 or #2 mutant were able to incorporate BrdU to significant levels, thereby indicating successful reactivation for these mutants (Figure 5C, 5D).

Reviewer #2 (Significance):

Conceptual advancement in showing ubiquitination could inhibit the mitochondrial import of a mitochondrial substrate. Proposes the inhibition is due to blockade of the interaction between import substrate and the TOM receptors. Remarkably shows blockade of ubiquitination could confer function to disease mutant-import substrates.

Mitochondrial, ubiquitin and cell biologists would be interested in these findings.

My background is in mitochondrial and ubiquitin biology thus was able to provide specific comments for their experiments.

Response:

We thank the reviewer for the positive comments.

Reviewer #3 (Evidence, reproducibility and clarity):

Hussain et al., report new mechanistic insights into the process of PolγA entry into mitochondria and how this is defective in a subset of disease-causing PolγA human mutations. Through a series of cell-free and cell-based experiments, the author report that the mitochondrial E3 ubiquitin ligase MITOL binds and ubiquitinates PolγA at lysine-1060, both leading to its degradation and decreasing PolγA's interaction with Tom20. This in turn reduces the ability of PolγA to enter the mitochondria. The authors also find that a subset of disease-causing PolγA mutations are excessively ubiquitinated at lysine-1060 which impacts its ability to enter the mitochondria. Mutation of the lysine-1060 within these disease-causing mutants decreases ubiquitination, allows PolγA entry into the mitochondria and rescues PolγA function.

- Are the key conclusions convincing?

While the main conclusions of the study are convincing, there are some significant concerns that should be addressed to improve the manuscript and make the outcomes more convincing.

- Should the authors qualify some of their claims as preliminary or speculative, or remove them altogether?

No, but further clarification of their claims (outlined below) would improve the manuscript.

- Would additional experiments be essential to support the claims of the paper? Request additional experiments only where necessary for the paper as it is, and do not ask authors to open new lines of experimentation.

Response:

We thank the reviewer for the positive comments.

Addressing the following would be important in supporting the claims of the paper -

Major query #1

Do the other Ubiquitin E3 Ligases bind and ubiquitinate PolγA? It appears that overexpression of Parkin increases the expression levels of PolγA (Figure 1A). Why?

Response:

This is a very interesting question. We do not think that other mitochondrial E3 ligases ubiquitylate PolγA and cause its degradation. This is because expression of the other mitochondrial E3 ligases did not decrease the levels of PolγA. Overexpression of only MITOL degraded PolγA (Figure 1A).

The E3 ubiquitin ligase Parkin has been shown to assemble multiple chains including K6-, K11-, K48- and K63-linked chains (PMID: 25284222). It is well known that ubiquitylation via K63 linkage stabilizes the levels of its substrates and enhances their functioning in multiple signalling cascades (reviewed in PMID: 28011818). Hence it is possible Perkin may also ubiquitylate PolγA by K63-ubiquitylation and thereby stabilize it. However except

stabilization of PolγA (seen in Figure 1A) we do not have any direct evidence in favour of such a hypothesis. We feel that investigating this question falls outside the ambit of this manuscript.

Major query #2

In-text description of Figure 2E = "HUWE1, known to ubiquitylate its substrate via K6-linakge (Michel et al., 2017), could not stabilize the levels of PolγA (Figure 2E), thereby indicating the specificity of MITOL towards PolγA ubiquitylation.". To justify the use of this statement, it assumes that HUWE1 binds and ubiquitinates PolγA. Does HUWE1 bind and ubiquitinate PolγA via K6-linkage? Please clarify.

Response:

In Figure S2A, ablation of another E3 ligase HUWE1 could not stabilize the levels of PolγA. This is in direct contrast with the effect on PolγA due to ablation of MITOL. Hence unlike MITOL, HUWE1 is not targeting PolγA for degradation. Hence the question of determining whether HUWE1 binds and ubiquitylates PolγA did not arise.

Major query #3

For Fig 3H and 3I, it's important to show equal expression of the Tom20-GST construct in "Ub-PolγA" and "Non Ub-PolγA".

Response:

We thank the reviewer for the suggestion. Coomassie gels showing equal expression of the Tom20-GST have been provided in the revised version of the manuscript (Figure 3H, 3I).

Major query #4

Figure S4B & S4C = The FLAG staining shown in Figure S4B of PEO #1 & #2 is highly saturated and therefore difficult to interpret - it also appears the expression levels are higher in these groups versus the others. A blot showing equal expression level in U2OS cells would be very beneficial here.

Response:

We agree with the reviewer's interpretation of the data. We also agree that the confocal images for PEO #1 and #2 in Figure S4B of the original version were over-saturated. However we have removed Figure S4B and S4C from the revised version of the manuscript.

Major query #5

The authors state "We also wanted to determine whether insoluble proteins encoded by PEO patients #1 and #2 had any aberrant localization." And also "confocal analysis indicated increased formation of aggregate-like structures when PEO patient mutants #1 and #2 were expressed." These statements require further clarification for the following reasons –

a. Flag-tagged PEO patient mutants #1 and #2 still appear mitochondrial when compared to cytochrome c staining.

b. How have these FLAG-positive puncta been defined as aggregates? The FLAG staining appears brighter, but which FLAG-positive puncta correspond to the aggregates in the figure? This is concerning as in the Discussion, this specific statement was made - "probably form aggregate-like structures (Figure S4A-S4C).". So clarification on this point is important.

Response:

We agree with the reviewer's viewpoint. We agree that quantitatively defining aggregates is difficult, if not impossible in the experiments which we had done. Moreover in normal confocal imaging it is impossible to discern whether the signal is on the surface of the mitochondria or within the mitochondrial matrix. We do not have access to super-resolution microscope. Hence we have removed Figure S4B and S4C from the revised version of the manuscript.

- Are the suggested experiments realistic in terms of time and resources? It would help if you could add an estimated cost and time investment for substantial experiments.

Yes.

- Are the data and the methods presented in such a way that they can be reproduced?

Yes, although the Methods section requires the addition of how the immunoblotting was performed, and also a section on statistical analysis.

Response:

We provided description of how the immunoblotting was performed (page 22), and also included a section on statistical analysis (page 23) in the revised version of the manuscript.

- Are the experiments adequately replicated and statistical analysis adequate?

Replication - According to the figure legends, each experiment has been replicated at least three times, which is adequate for cell-free and cell-based systems.

Statistical analysis - the statistical analysis presented in this study (only using the 'Student's t test') is technically incorrect. A 'Student's t test' should only be used for determining the statistical significance between two sets of data or groups. If two or more groups are compared to one group (for example, PolγA WT to PEO #1 and PEO #2 in figure 4H), then a one-way ANOVA is required. If two or more groups are compared to over time (for example, Figure 1I where siControl is compared to siMITOL over CHX treatment times), then a two-way ANOVA is required. Please repeat the statistical analysis using the required statistical tests. In addition, statistical analysis of the quantification of immunoblotting intensities, such as in Figure 1A is required.

Response:

We appreciate the reviewer's query. We have re-done the statistical analysis of all the quantifications. Almost in all cases either One-way ANOVA or Two-way ANOVA have been used. The statistical analysis performed in each study has been listed in Table S6. A section has been added in the methods section under the heading "statistical analysis" (page 23) – where we have indicated that all data have been presented as mean ± SD. * $p < 0.05$, ** $p < 0.01$, *** $p < 0.001$, **** $p < 0.001$.

Minor comments:

-Specific experimental issues that are easily addressable.

Minor query #1

The quantification of many immunoblots are presented as a number on the blot, which as a reader, makes it difficult to determine what these numbers represent; ie, do the presented numbers on the blots represent the average value of the three experiments or just the single experiment presented in the figure? If only once, additional biological replicates would be required so that the quantification can be presented in format such as a dot plots/bar graphs, with error bars depicting the standard deviation of the data.

Response:

We have positively responded to the suggestion of the reviewer. Hence we have provided quantification in a bar plot for a number of experiments involving immunoblots. Further we have done the statistical analysis of all these quantifications. The statistical analysis in each case has been listed in Table S6. We have indicated in every figure legend the number of times each experiment was done.

Minor query #2

Figure S1A = Endogenous colocalization of MITOL and PolγA is presented. The representative DAPI image shows small DAPI-positive puncta in the cytoplasm, not typical of DAPI staining restricted to the nucleus. Why are these puncta present? Could this be bleed through from other antibody staining or be mycoplasma contamination? 'The percentage of cells (n=200) showing colocalization has been indicated.' What is considered 'colocalization' in this context? A single puncta or all puncta colocalising? Clarification on this is necessary.

Response:

This query refers to Figure S1C of the revised version. We have checked the images and there is no bleed through. The cells have been tested for mycoplasma and it was always negative. These puncta are the mitochondrial nucleoids where mitochondrial replication is known to occur (PMIDs: 23821750, 28680532). In the mitochondrial nucleoids DAPI staining is observed (PMIDs:) as they contain mitochondrial DNA (PMID: 23821750, 28680532, 27371858).

We have re-quantitated the extent of colocalization by determining colocalization factor. Colocalization factor takes into account both the fraction of cells having colocalization and the fraction of foci colocalized per cells. The information regarding colocalization factor has been incorporated in the materials and methods (page 18).

Minor query #3

The presented images also look like there has been significant post-acquisition processing (especially MITOL), and look artificial in nature. Replacing these images with another representative image without this post-acquisition processing would improve the presentation.

Response:

We have presented alternate images for Figure S1C in the revised version of the manuscript.
.

Minor query #4

Figure 1C = the change is not convincing, would recommend the presentation of another blot

Response:

We presented an alternate biological replicate experiment in Figure 1C of the revised version of the manuscript.

Minor query #5

Figure 2C = Further explanation on R versus O Ub mutants would be beneficial in the figure legend.

Response:

This refers to Figure 2D of the revised version of the manuscript. We have provided the explanation of “R” vs “O” mutants in the figure legend of the revised version (page 35).

Minor query #6

Figure 4A & B = PEO #3 is presented as a cut blot, separate from the WT. Was the WT & PEO #3 developed on the same blot or on independent blots? This should be made clear as quantification of these bands and comparisons to WT are presented in 4B.

Response:

We appreciate the reviewer’s comment. The experiment involving WT vs PEO #3 was done separately and hence in separate blots. This explanation has been provided in the figure legend of Figure 4A in the revised version of the manuscript (page 38).

Minor query #7

Clarify what is meant by 'early time-points' for reference to figure 4D.

Response:

We appreciate the reviewer's comment. To determine the difference in the rate of ubiquitylation and degradation between wildtype and mutant PolγA (as seen in Figure 4E of the revised version), lysates were made 12 hrs post-transfection. This included the period of MG132 (10µM) treatment which was carried out for 5 hrs immediately prior to the preparation of the lysates. This explanation has been provided in the Methods section in the revised version of the manuscript (page 17).

Minor query #8

Figure 5C = "The K1060R counterparts of the PEO patient mutants #1 and #2 entered the mitochondria with efficiencies which were comparable to PolγA WT". Is there a statistically significant difference between the PolγA WT and PEO mutants? Given that the quantification of WT versus PEO mutants shows an average that is approx. 50-60% lower than WT, statistical analysis would be beneficial here to justify the use of 'comparable'.

Response:

We appreciate the reviewer's comment. Responding to the query we have now removed the term "comparable". Now we simply state "The K1060R counterparts of the PEO mutants #1 and #2 entered the mitochondria with efficiencies which were significantly more than that observed for PEO mutants #1 and #2 themselves" (page 13).

-Are prior studies referenced appropriately?
The field appears to be referenced well in this study.

-Are the text and figures clear and accurate?

Text formatting- All required sections are present. However, to improve their Methods section, the authors need to include a section on how they performed their immunoblotting (or provide a suitable reference) and also provide a section on their statistical analysis. The references to specific figure numbers are usually not required in the Discussion section.

Response:

In the revised version we have provided description of how the immunoblotting was performed (Method section, page 22), and also provided a section on statistical analysis (Method section, page 23). Further the figure numbers have been removed from the Discussion section.

Figure formatting

-

1) The figures and text within the figures appear quite pixelated. While this does not impact the data itself, simply exporting the figures with a higher dpi will improve their readability. Although I understand this may be the result of file compression when submitting to the journal.

Response:

We have exported the figures at a higher dpi in the revised version. We thank the reviewer for pointing this out.

2) As raised previously, the immunofluorescence images presented in this study appear very pixelated, with some images appearing highly saturated (for example, the FLAG staining in the PEO Mutants #1 & #2 of Figure S4B). This makes the images difficult to interpret, and replacing these with images that are not saturated would significantly improve this.

Response:

We agree with the reviewer's viewpoint. We agree that quantitatively defining aggregates is difficult, if not impossible. Moreover in normal confocal imaging it is impossible to discern whether the signal is on the surface of the mitochondria or within the mitochondrial matrix. We do not have access to super-resolution microscope. Hence have removed the original Figure S4B and S4C from the revised version of the manuscript.

Reviewer #3 (Significance):

The work builds on two previous studies published in Molecular Cell - PMID: 32142684 & PMID: 32142685, which show that ubiquitination of proteins can influence their import into the mitochondria. This study further builds on these two studies by identifying PolγA as a protein that is ubiquitinated by MITOL to control its mitochondrial import. The significance in the study is in the discovery of a potential molecular mechanism of disease caused by specific PolγA mutations in humans, which links excessive ubiquitination of PolγA to defective mitochondrial import and errors in mitochondrial DNA. The work will largely appeal to researchers interested in mitochondrial DNA replication, regulation of protein import into mitochondria.

The reviewer has expertise in mitochondrial biochemistry and disease, trafficking and cell signalling.

Response:

We thank the reviewer for the positive comments.
