# Supplementary material for: MITOL-dependent ubiquitylation negatively regulates the entry of PolγA into mitochondria
Source: PLoS Biol. 2021 Mar 3;19(3):e3001139. doi: 10.1371/journal.pbio.3001139 (PMC7959396; doi:10.1371/journal.pbio.3001139)
Supplement: Attachment — Submitted filename: 108311_1_rebuttal_1828717_qnnc0g.docx [file pbio.3001139.s015.docx]

**Response to reviewer's comments**

**Reviewer #1 (Evidence, reproducibility and clarity (Required)):**

****Summary****

In this manuscript that authors have dissected how a key mitochondrial replication protein PolγA is ubiquitylated on the mitochondrial outer membrane by MITOL, an E3 ligase present on the mitochondrial outer membrane. Using a number of in vivo and in vitro assays the authors demonstrate that MITOL ubiquitylates PolγA at specific residues using K6 linkage. Only non-ubiquitylated or hypo-ubiquitylated PolγA can enter mitochondrial matrix and this enhances their functions during mtDNA replication. Further mutant PolγA variants present in some of the PEO patients are hyper-ubiquitylated by MITOL. This results in formation of "aggregate like structures" for mutant PolγA which cannot enter mitochondria and hence is less effective during mtDNA replication. Finally two independent lines of evidence show that non-functional PolγA patient mutants can be reactivated. The results are compelling and the relevant experimental controls are present. However certain additional explanations are needed and some experiments need to be performed to further strengthen the manuscript.

**Response:**

We thank the reviewer for the positive comments.

****Major Points****

**Major query #1**

Overall the concept is that the mere presence of mitochondrial localization signal is not sufficient for proteins to enter into the mitochondrial matrix and finer regulatory control like ubiquitylation is necessary. This should be more elaborated in the Discussion.

**Response:**

We completely agree with the reviewer. We have included this pertinent point in the Discussion in the next version manuscript (please see marked up copy, page 13).

**Major query #2**

Figure 2 and others: Ubiquitylation of PolγA were observed by ladder formation with anti-PolγA antibody, but it seems that the amounts of substrate (PolγA) were not equal. It looks like WB data probed with anti-ubiquitin antibody. In contrast, the same amounts of 35S PolγA are almost equal. The authors should explain this difference and the reason why no ladder formation of 35S PolγA was observed.

**Response:**

We would like to point it out that all the blots for in vitro ubiquitylation (Figure 2C, 2F, S2B, S2C, S2E) were carried out with anti-PolγA antibody and not with anti-ubiquitin antibody. The amount of substrate taken was equal as seen by the 35^S^ PolγA autoradiogram. Infact every time after the westerns were, done the same blot was exposed to get the 35^S^ PolγA autoradiogram.

Further, it is to be noted that PolγA ubiquitylated smears were not observed upon autoradiography. This was due to the fact that the SDS-PAGE gels had to be subjected to fluorography (as S^35^ Methionine is a weak beta emitter), the gels dried at 65°C for 3-4 hrs, before being exposed for autoradiography. The process of fluorography and/or the heating the gels during the drying process degraded the ubiquitylated products for a high molecular weight substrate like PolGA. This explanation has been provided in the Materials and Methods section of the next version of the manuscript (please see marked up copy, page 17).

**Major query #3**

Figure 2C: The authors showed that, in vitro ubiquitination assay, MITOL ubiquitinates PolγA in a K6-specific manner. In my experience, in vitro, E3 ligases randomly add ubiquitin chains on substrates. It is speculated that E2 and other factors may be involved in the determining of pattern of ubiquitin chain formation in cells. Is this K6-specific Ub in vitro due to the specific property of PolγA? This should be more elaborated in the Discussion.

**Response:**

We thank the reviewer for this query.

In “R” ubiquitin mutants, only a particular lysine residue in ubiquitin is mutated while in “O” ubiquitin mutants only one lysine is present on ubiquitin and all other six lysines on ubiquitin are mutated. This information has been provided in the Materials and Methods section of the next version of the manuscript (please see marked up copy, page 17).

We believe that the K6-specific ubiquitylation in vitro is a specific property of PolγA. We came to this conclusion because except K6R, for none of the other “R” mutants of PolγA there is a loss of in vitro ubiquitylation of PolγA. This indicated that loss of ubiquitylation on PolγA is linked to the lack of lysine residue at K6 position of ubiquitin. Subsequently when we used the K6O mutant (where only the lysine at 6^th^ position on ubiquitin is present and all other lysines are mutated to alanines) in the in vitro ubiquitylation assay, there is robust ubiquitylation of PolγA. It is to be noted that usage of K48O or K63O ubiquitin mutants did not lead to PolγA ubiquitylation (as in both K48O or K63O ubiquitin mutants, the lysine at the 6^th^ position in ubiquitin has been mutated to alanine). We have added the above explanation in the Result section in new version of the manuscript (Please see result section of the marked up copy page 8).

**Major query #4**

Figure 4E: using Southwestern and slot blot analysis authors show that some of the PEO mutants do not incorporate BrDU and hence they are presumably defective in mtDNA replication. Will the same PEO mutants behave similarly when used for in vitro exonuclease and polymerase assays?

**Response:**

We thank the reviewer for the comments.

We will carry out in vitro exonuclease and polymerase assays with wildtype PolγA and the four tested PEO mutants. This data can be incorporated in the revised of the manuscript.

**Major query #5**

How does non-ubiquitylated PolγA which enters into the mitochondrial matrix undergo turnover? Are these proteins again exported out to the mitochondrial surfaces to be targeted by MITOL? Or do these proteins undergo turnover inside mitochondrial matrix?

**Response:**

We thank the reviewer for the very pertinent question. It is possible that PolγA which enters into the mitochondria can undergo turnover in two ways: either exported out again of the mitochondria (as postulated for other proteins PMIDs: 14731958, **11251115,** 9482871) or becoming a target of mitochondrial proteases (as reviewed in PMID: 28589125, 32075415). While this question is very interesting, it falls outside the purview of this manuscript.

**Major query #6**

Two of the four PEO mutants do not form aggregates, are not degraded by MITOL and enter into the mitochondria with equal efficiency as wildtype PolγA. What is the reason of discrimination between the PEO mutants which allows some mutants but not others to be a MITOL target?

**Response:**

We thank the reviewer for this very interesting scientific comment.

In this case we are dealing with single point mutations which cause drastic changes in disease causing phenotype – one class of PolγA mutants (PEO mutants #1 and #2) form aggregates which remain outside the mitochondria. The second class of PolγA mutants (PEO mutants #3 and #4) go inside the mitochondria, much like wildtype PolγA.

We do not know whether we can solve this problem within the period usually given for the revision by the journals. However we will try the following two experimental approaches:

A. The crystal structure of full-length wildtype PolγA is known (PDB: 4LT6 and 3IKM). We propose to carry out in silico docking studies to understand whether the four PEO mutants self-associate with each other and thereby probably form aggregates. Wildtype PolγA which does not form a dimer will serve as a control. This line of analysis may help us to understand why two of the four PEO mutants do not form aggregates, are not degraded by MITOL.

B. We will co-express Flag tagged wildtype PolγA and each of the four PEO mutants with myc-tagged MITOL. It is expected that MITOL will differentially ubiquitylate the five different PolγA proteins. Wildtype PolγA, PEO mutants #3, PEO mutants #4 will be ubiquitylated less while PEO mutants #1 and PEO mutants #2 will be ubiquitylated more. These different PolγA will be immunoprecipitated with anti-Flag beads and purified Flag tagged proteins obtained post-elution with Flag peptide. These proteins will be used to detect their Circular Dichroism (CD) profile to detect any conformation change, if any, due to differential ubiquitylation.

If successful, the data can be incorporated in the revised version of the manuscript.

****Minor Points****

**Minor query #1**

Figure 4: They showed the ubiquitination and proteasomal degradation of PEO mutants by MITOL overexpression. It is better to show accumulations and decreased mitochondrial import of PEO mutants in MITOL-knockdown cells. If possible, CHX assay should be performed.

**Response:**

We will use HeLa cells in which MITOL has been ablated by shRNA. In these cells we propose to overexpress equal amount of Flag tagged WT and PEO mutants, isolate the mitoplast and check the levels of imported PolγA proteins into the organelle. If successful, this data can be incorporated in the revised version of the manuscript.

This is a problem of import for the PolγA mutant proteins into the mitochondria, We feel that CHX experiment (which measures the half-life of the proteins) will not be informative in MITOL deficient background. Hence CHX experiment will not be attempted.

**Minor query #2**

The authors should speculate the fate of the cells which accumulate the PEO patient mutants. If possible, a live cell experiment should be performed to know whether the cells showing accumulation of these mutant proteins survive in subsequent generations.

**Response:**

We appreciate the reviewer’s comments. This line of study is part of an active research project in the lab whereby stable lines expressing PolγA wildtype and the four mutants are in the process of being generated. These stable lines will be characterized using live cell microscopy. However this component will be outside the purview of the present manuscript.

**Minor query #3**

It is very interesting to see if USP30 suppresses PolγA ubiquitination by MITOL. If possible, please check it.

**Response:**

We appreciate the reviewer’s query. We will use both in vitro and in vivo assays to determine whether USP30 suppresses PolγA ubiquitination by MITOL. In vivo, we will overexpress or ablate the level of USP30 to determine whether alteration of USP30 levels affect PolγA protein levels and its subsequent ubiquitylation. In vitro we propose to carry out in vitro ubiquitylation of PolγA by MITOL in presence or absence of recombinant USP30. If successful, this data can be incorporated in the revised version of the manuscript.

**Reviewer #1 (Significance (Required):**

****Nature and significance of the advance.****

This work reveals novel mechanisms in mitochondrial DNA replication and quality control, and suggests the molecular pathology of PEO.

****Compare to existing published knowledge.****

This paper basically follows the concept of a previous paper that "Dynamic Regulation of Mitochondrial Import by the Ubiquitin System" published in Mol Cell 2020, and shows further development in the regulation of mitochondrial import system.

****Audience.****

Relatively broad

****My expertise.****

Mitochondrial biology, especially ubiquitin-mediated signaling on the mitochondrial outer membrane

**Response:**

We thank the reviewer for the positive comments.

**Reviewer #2 (Evidence, reproducibility and clarity (Required):**

This is an interesting manuscript by Hussain et al. who proposes that mitochondrial-disease associated mutations in PolgA lead to defects in their mitochondrial import. Specifically, mitochondrial-surface attached ligase MITOL promotes ubiquitination of PolgA, impairing its import. The "non-ubiquitinatable" PolgA are imported more efficiently, leading to improved PolgA function. The manuscript builds upon the previous data on quality control pathways at the mitochondrial translocon and suggests engagement of these pathways on mutated mitochondrial proteins limit their import and function. Remarkably, mutating the lysine that is ubiquitinated in mutant PolgA restores its import and function. The biochemical data presented extensively examines the ubiquitination of PolgA by MITOL. Additional experiments providing technical controls and linking the impact of ubiquitination of mutants to March5 and mitochondrial function should further improve the manuscript.

The studies shown could be improved by providing the quantitation of immunoblotting experiments as suggested below. The additional experiments suggested are mainly aimed at showing that endogenous MITOL in cells carry out the functions as hypothesized by the authors.

**Response:**

We thank the reviewer for the positive comments.

**Major query #1**

The authors should also improve statistical comparisons by using ANOVA and post-hoc tests (in multiple comparisons) rather than using multiple t-tests.

**Response:**

We appreciate the reviewer’s query. We will improve the statistical comparisons by using ANOVA and post-hoc tests (in multiple comparisons) rather than using multiple t-tests. The improved statistical analysis will be incorporated in the revised version of the manuscript.

**Major query #2**

Does mRNA levels of PolgA change upon MITOL over expression or knockdown?

**Response:**

We will carry out the suggested experiment. We will overexpress or ablate MITOL and check the mRNA levels of PolGA. This data can be incorporated in the revised version of the manuscript.

**Major query #3**

In experiments where immunoblotting was performed >3 times, please show quantifications in a bar plot especially in experiments where the change in immunoblot signal is small e.g. 1C, control vs MG132; 2D, control vs MITOL etc.

**Response:**

We will provide the requested bar plots for the quantitation. This will be done in all experiments where there is change in the immunoblot signal. All quantifications will be incorporated in the revised version of the manuscript.

**Major query #4**

Please provide additional markers in Fig. 1E-G to show overall mitochondrial proteome is intact. For example, show levels of proteins studied in Fig 1B in these MITOL-depletion experiments.

**Response:**

We appreciate the reviewer’s query. We will provide additional markers in Figure 1E, 1F, 1G to show that the mitochondrial proteome is intact when MITOL is depleted. This data can be incorporated in the revised version of the manuscript.

**Major query #5**

Why does the "non-ubiquitinatable" K1060R mutant not stabilized (increased protein levels) compared to other mutants in Fig 2G?

**Response:**

We appreciate the reviewer’s concern. This is to do with the experimental conditions – the time-point when the lysates were made. The time point used for this experiment (24 hrs post-transfection) was perfect to detect robust ubiquitylation (or its loss) (as seen in the IP/Western blot). However at a later time point (36 hrs post-transfection) increased stabilization was observed for K1060 mutant – this western data will be provided in the next version of the manuscript.

**Major query #6**

Does knockdown of MITOL eliminate ubiquitination of PolgA?

**Response:**

We propose to address the reviewer’s query. We will use HeLa cells in which MITOL has been ablated by shRNA or use HEK293T cells in which MITOL is depleted by siRNA treatment. PolGA will be immunoprecipitated from both conditions and checked for its ubiquitylation. This data can be incorporated in the revised version of the manuscript.

**Major query #7**

Does ubiquitination additionally alter interaction with TOM70 receptor (a receptor that is responsible for importing aggregation-protein/hydrophobic substrates)?

**Response:**

While we appreciate the reviewer’s question – we think that this question will be outside the purview of this manuscript.

**Major query #8**

What are the consequences of K6-mediated ubiquitination on PolgA? Are the effects described in Fig1 due to K6-ubiquitination? Does inhibition of proteasomes stabilize the K6-linked PolgA substrate?

**Response:**

While we appreciate the reviewer’s question – repeating the entire set of experiments in Figure 1 with specific emphasis on K6-ubiquitylation will not be possible. It is implied that the effects observed in Figure 1 are due to K6-ubiquitylation on PolgA.

**Major query #9A**

Did the authors use MITOL-ubiquitinated substrate in the import reactions shown in Fig 3? This is suggested in the methods but not clear in the main text.

**Response:**

Yes – we had used ubiquitylated PolgA. We have clarified this in the main text of the Result section (page 9) and also in the figure legend (page 31) of the marked up copy.

**Major query #9B**

If substrates that were in vitro ubiquitinated were used for import reactions, please show the efficiency of ubiquitination on Western blots.

**Response:**

We will provide western blots showing that PolgA was ubiquitylated in the supplementary data section. This data can be incorporated in the revised version of the manuscript.

**Major query #9C**

What fraction of presented substrate is ubiquitinated in Fig 3C?

**Response:**

A significant amount of the substrate is ubiquitylated – as will be apparent from the western blot which will be supplied. However is difficult to quantitate the exact percentage of ubiquitylation without quantitative mass spec analysis. We think this line of experimentation is outside the purview of this manuscript.

**Major query #9D**

If PolgA substrate is ubiquitinated, why does it run at the same molecular weight as non-Ub? Input for the Ub PolgA should also be shown in this panel.

**Response:**

It should be noted that all import assays were assayed by autoradiography. It is obvious that PolγA ubiquitylated smears were not observed upon autoradiography. This was due to the fact that the SDS-PAGE gels had to be subjected to fluorography (as S^35^ Methionine is a weak beta emitter), the gels dried at 65°C for 3-4 hrs, before being exposed for autoradiography. The process of fluorography and/or the heating the gels during the drying process degraded the ubiquitylated products for a high molecular weight substrate like PolGA. This explanation has been provided in the Materials and Methods section of the next version of the manuscript (please see marked up copy, page 17).

**Major query #10**

Are the PEO patient 1 and 2 PolgA Ub-ed by K6 linkage?

**Response:**

This is an interesting query. Will carry out in vitro ubiquitylation assays with PolgA wildtype vs the two PEO mutants (mutant #1 and 2). We propose to use wildtype ubiquitin, ubiquitin K6R and K6O mutants in this experiment. If successful, this data can be incorporated in the revised version of the manuscript.

**Major query #11**

Does knockdown of MITOL reduce ubiquitination and promote mitochondrial import of mutant PEO patient 1 and 2 PolgA in cells?

**Response:**

We appreciate the reviewer’s query. We propose to use HeLa shMITOL cells to deduce whether knockdown of MITOL will reduce PolgA ubiquitylation. We also propose to overexpress equal amount of Flag tagged WT and PEO mutants, isolate the mitoplast and check the levels of imported PolγA proteins into the organelle. If successful, this data can be incorporated in the revised version of the manuscript.

**Major query #12**

Does MITOL knockdown rescue the BrdU incorporation defects?

**Response:**

We appreciate the reviewer’s question. We will use HeLa shControl and HeLa shMITOL cells and carry out Southwestern analysis and slot blot assays. If successful, this data can be incorporated in the revised version of the manuscript.

**Reviewer #2 (Significance (Required):**

Conceptual advancement in showing ubiquitination could inhibit the mitochondrial import of a mitochondrial substrate. Proposes the inhibition is due to blockade of the interaction between import substrate and the TOM receptors. Remarkably shows blockade of ubiquitination could confer function to disease mutant-import substrates.

Mitochondrial, ubiquitin and cell biologists would be interested in these findings.

My background is in mitochondrial and ubiquitin biology thus was able to provide specific comments for their experiments.

**Response:**

We thank the reviewer for the positive comments.

**Reviewer #3 (Evidence, reproducibility and clarity (Required):**

Hussain et al., report new mechanistic insights into the process of PolγA entry into mitochondria and how this is defective in a subset of disease-causing PolγA human mutations. Through a series of cell-free and cell-based experiments, the author report that the mitochondrial E3 ubiquitin ligase MITOL binds and ubiquitinates PolγA at lysine-1060, both leading to its degradation and decreasing PolγA's interaction with Tom20. This in turn reduces the ability of PolγA to enter the mitochondria. The authors also find that a subset of disease-causing PolγA mutations are excessively ubiquitinated at lysine-1060 which impacts its ability to enter the mitochondria. Mutation of the lysine-1060 within these disease-causing mutants decreases ubiquitination, allows PolγA entry into the mitochondria and rescues PolγA function.

*- Are the key conclusions convincing?*

While the main conclusions of the study are convincing, there are some significant concerns that should be addressed to improve the manuscript and make the outcomes more convincing.

*- Should the authors qualify some of their claims as preliminary or speculative, or remove them altogether?*

No, but further clarification of their claims (outlined below) would improve the manuscript.

*- Would additional experiments be essential to support the claims of the paper? Request additional experiments only where necessary for the paper as it is, and do not ask authors to open new lines of experimentation.*

**Response:**

We thank the reviewer for the positive comments.

Addressing the following would be important in supporting the claims of the paper -

**Major query #1**

Do the other Ubiquitin E3 Ligases bind and ubiquitinate PolγA? It appears that overexpression of Parkin increases the expression levels of PolγA (Figure 1A). Why?

**Response:**

This is a very interesting question. We do not think that other mitochondrial E3 ligases ubiquitylate PolγA and cause its degradation. This is because expression of these E3 ligases do not decrease the levels of PolγA (Figure 1A). However it is possible Perkin may ubiquitylate PolγA by K63-ubiquitylation and thereby stabilize it. However we do not have any evidence in favour of such a hypothesis. We feel that this question falls outside the ambit of this manuscript.

**Major query #2**

In-text description of Figure 2E = "HUWE1, known to ubiquitylate its substrate via K6-linakge (Michel et al., 2017), could not stabilize the levels of PolγA (Figure 2E), thereby indicating the specificity of MITOL towards PolγA ubiquitylation.". To justify the use of this statement, it assumes that HUWE1 binds and ubiquitinates PolγA. Does HUWE1 bind and ubiquitinate PolγA via K6-linkage? Please clarify.

**Response:**

In Figure 2E, **ablation of** another E3 ligase HUWE1 could not stabilize the levels of PolγA. Hence it means that HUWE1 is not targeting PolγA for degradation. Hence the question of determining whether HUWE1 binds and ubiquitylates PolγA did not arise.

**Major query #3**

For Fig 3H and 3I, it’s important to show equal expression of the Tom20-GST construct in "Ub-PolγA" and "Non Ub-PolγA".

**Response:**

We thank the reviewer for the suggestion. Coomassie gels showing equal expression of the Tom20-GST will be provided in the revised version of the manuscript.

**Major query #4**

Figure S4B & S4C = The FLAG staining shown in Figure S4B of PEO #1 & #2 is highly saturated and therefore difficult to interpret - it also appears the expression levels are higher in these groups versus the others. A blot showing equal expression level in U2OS cells would be very beneficial here.

**Response:**

We agree with the reviewer’s interpretation of the data. All the PEO mutants were expressed at the same level. A blot showing equal expression level in U2OS cells will be provided as a control for Figure S4A in the revised version of the manuscript.

We also agree that the confocal images for PEO #1 and #2 in Figure S4B are over saturated. Hence we propose to remove Figure S4B and S4C from the next version of the manuscript (please also see comment below for Major query #5).

**Major query #5**

The authors state "We also wanted to determine whether insoluble proteins encoded by PEO patients #1 and #2 had any aberrant localization." And also "confocal analysis indicated increased formation of aggregate-like structures when PEO patient mutants #1 and #2 were expressed." These statements require further clarification for the following reasons –

a. Flag-tagged PEO patient mutants #1 and #2 still appear mitochondrial when compared to cytochrome c staining.

b. How have these FLAG-positive puncta been defined as aggregates? The FLAG staining appears brighter, but which FLAG-positive puncta correspond to the aggregates in the figure?

This is concerning as in the Discussion, this specific statement was made - "probably form aggregate-like structures (Figure S4A-S4C).". So clarification on this point is important.

**Response:**

We agree with the reviewer’s viewpoint. We agree that quantitatively defining aggregates is difficult, if not impossible in the present experiment. Moreover in normal confocal imaging it is impossible to discern whether the signal is on the surface of the mitochondria or within the mitochondrial matrix. We do not have access to super-resolution microscope. Hence we propose to remove Figure S4B and S4C from the next version of the manuscript.

*- Are the suggested experiments realistic in terms of time and resources? It would help if you could add an estimated cost and time investment for substantial experiments.*

Yes.

*- Are the data and the methods presented in such a way that they can be reproduced?*

Yes, although the Methods section requires the addition of how the immunoblotting was performed, and also a section on statistical analysis.

**Response:**

We will provide description of how the immunoblotting was performed, and also a section on statistical analysis. These additional writeups can be incorporated in the revised version of the manuscript.

*- Are the experiments adequately replicated and statistical analysis adequate?*

Replication - According to the figure legends, each experiment has been replicated at least three times, which is adequate for cell-free and cell-based systems.

Statistical analysis - the statistical analysis presented in this study (only using the 'Student's t test') is technically incorrect. A 'Student's t test' should only be used for determining the statistical significance between two sets of data or groups. If two or more groups are compared to one group (for example, PolγA WT to PEO #1 and PEO #2 in figure 4H), then a one-way ANOVA is required. If two or more groups are compared to over time (for example, Figure 1I where siControl is compared to siMITOL over CHX treatment times), then a two-way ANOVA is required. Please repeat the statistical analysis using the required statistical tests. In addition, statistical analysis of the quantification of immunoblotting intensities, such as in Figure 1A is required.

**Response:**

We appreciate the reviewer’s query. We will improve the statistical comparisons by using ANOVA and post-hoc tests (in multiple comparisons) rather than using multiple t-tests. Further we will provide the requested bar plot for the quantitation. This will be done in all experiments where there is change in the immunoblot signal. The improved statistical analysis will be incorporated in the revised version of the manuscript.

****Minor comments:****

*-Specific experimental issues that are easily addressable.*

**Minor query #1**

The quantification of many immunoblots are presented as a number on the blot, which as a reader, makes it difficult to determine what these numbers represent; ie, do the presented numbers on the blots represent the average value of the three experiments or just the single experiment presented in the figure? If only once, additional biological replicates would be required so that the quantification can be presented in format such as a dot plots/bar graphs, with error bars depicting the standard deviation of the data.

**Response:**

We will provide the requested bar plot for the quantitation. This will be done in all experiments where there is change in the immunoblot signal. These will be incorporated in the revised version of the manuscript.

**Minor query #2**

Figure S1A = Endogenous colocalization of MITOL and PolγA is presented. The representative DAPI image shows small DAPI-positive puncta in the cytoplasm, not typical of DAPI staining restricted to the nucleus. Why are these puncta present? Could this be bleed through from other antibody staining or be mycoplasma contamination?
'The percentage of cells (n=200) showing colocalization has been indicated.' What is considered 'colocalization' in this context? A single puncta or all puncta colocalising? Clarification on this is necessary.

**Response:**

We have checked the images and there is no bleed through. The cells have been tested for mycoplasma and it was always negative. These puncta are the mitochondrial nucleoids where mitochondrial replication is known to occur (PMIDs: 23821750, 28680532). The mitochondrial nucleoids DAPI staining is observed (PMIDs:) as they contain mitochondrial DNA (PMID: 23821750, 28680532, **27371858).**

**Minor query #3**

The presented images also look like there has been significant post-acquisition processing (especially MITOL), and look artificial in nature. Replacing these images with another representative image without this post-acquisition processing would improve the presentation.

**Response:**

We will present alternate image for Figure S1A The improved statistical analysis will be incorporated in the revised version of the manuscript.

**Minor query #4**

Figure 1C = the change is not convincing, would recommend the presentation of another blot

**Response:**

We will present an alternate biological replicate experiment in Figure 1C. This data can be incorporated in the revised version of the manuscript.

**Minor query #5**

Figure 2C = Further explanation on R versus O Ub mutants would be beneficial in the figure legend.

**Response:**

We have provided the explanation of “R” vs “O” mutants in the figure legend of Figure 2C in the revised version (please see marked up copy, page 30).

**Minor query #6**

Figure 4A & B = PEO #3 is presented as a cut blot, separate from the WT. Was the WT & PEO #3 developed on the same blot or on independent blots? This should be made clear as quantification of these bands and comparisons to WT are presented in 4B.

**Response:**

We appreciate the reviewer’s comment. The experiment involving WT vs PEO #3 was done separately and hence in separate blots. This explanation has been provided in the figure legend of Figure 4A in the revised version of the manuscript (please see marked up copy, page 33).

**Minor query #7**

Clarify what is meant by 'early time-points' for reference to figure 4D.

**Response:**

We appreciate the reviewer’s comment. To determine the difference in the rate of ubiquitylation and degradation between wildtype and mutant PolgA (as seen in Figure 4D), lysates were made 12 hrs post-transfection. This included the period of MG132 (10μM) treatment which was carried out for 5 hrs immediately prior to the preparation of the lysates. This explanation will be provided in the Methods section in the revised version of the manuscript (please see marked up copy, page 16).

**Minor query #8**

Figure 5C = "The K1060R counterparts of the PEO patient mutants #1 and #2 entered the mitochondria with efficiencies which were comparable to PolγA WT". Is there a statistically significant difference between the PolγA WT and PEO mutants? Given that the quantification of WT versus PEO mutants shows an average that is approx. 50-60% lower than WT, statistical analysis would be beneficial here to justify the use of 'comparable'.

**Response:**

We appreciate the reviewer’s comment. We will carry out appropriate statistical analysis and report whether there is a statistically significant difference between the PolγA WT and PEO mutants. This statistical analysis will be incorporated in the revised version of the manuscript.

*-Are prior studies referenced appropriately?*

The field appears to be referenced well in this study.

*-Are the text and figures clear and accurate?*

Text formatting- All required sections are present. However, to improve their Methods section, the authors need to include a section on how they performed their immunoblotting (or provide a suitable reference) and also provide a section on their statistical analysis. The references to specific figure numbers are usually not required in the Discussion section.

**Response:**

In the revised version we will provide description of how the immunoblotting was performed, and also a section on statistical analysis. However we prefer to keep the figure numbers in the Discussion.

Figure formatting -

1) The figures and text within the figures appear quite pixelated. While this does not impact the data itself, simply exporting the figures with a higher dpi will improve their readability. Although I understand this may be the result of file compression when submitting to the journal.

**Response:**

We will export the figures at a higher dpi in the revised version. We thank the reviewer for pointing this out.

2) As raised previously, the immunofluorescence images presented in this study appear very pixelated, with some images appearing highly saturated (for example, the FLAG staining in the PEO Mutants #1 & #2 of Figure S4B). This makes the images difficult to interpret, and replacing these with images that are not saturated would significantly improve this.

**Response:**

We agree with the reviewer’s viewpoint. We agree that quantitatively defining aggregates is difficult, if not impossible. Moreover in normal confocal imaging it is impossible to discern whether the signal is on the surface of the mitochondria or within the mitochondrial matrix. We do not have access to super-resolution microscope. Hence we propose to remove Figure S4B and S4C from the next version of the manuscript.

Reviewer #3 (Significance (Required):

The work builds on two previous studies published in Molecular Cell - PMID: 32142684 & PMID: 32142685, which show that ubiquitination of proteins can influence their import into the mitochondria. This study further builds on these two studies by identifying PolγA as a protein that is ubiquitinated by MITOL to control its mitochondrial import. The significance in the study is in the discovery of a potential molecular mechanism of disease caused by specific PolγA mutations in humans, which links excessive ubiquitination of PolγA to defective mitochondrial import and errors in mitochondrial DNA. The work will largely appeal to researchers interested in mitochondrial DNA replication, regulation of protein import into mitochondria.

The reviewer has expertise in mitochondrial biochemistry and disease, trafficking and cell signalling.

**Response:**

We thank the reviewer for the positive comments.
