# Supplementary material for: MITOL-dependent ubiquitylation negatively regulates the entry of PolγA into mitochondria
Source: PLoS Biol. 2021 Mar 3;19(3):e3001139. doi: 10.1371/journal.pbio.3001139 (PMC7959396; doi:10.1371/journal.pbio.3001139)
Supplement: Attachment — Submitted filename: Response to reviewers comments.docx [file pbio.3001139.s018.docx]

**Response to reviewers' comments**

**Reviewer #1:**

The authors have made excellent efforts resolving the raised issues. I have no further comments to add.

**Reviewer #2:**

The authors adequately addressed the major comments raised by the reviewers. The additional data on functional effects of MITOL knockdown on mitochondrial import further strengthened the manuscript. The manuscript should be in good shape for publication in Plos Biology.

**Reviewer #3:**

I am satisfied with the author's response to my comments.

**Common response to all the reviewers:**

We thank all the reviewers for their positive comments.
